# System dynamics model for improving the robustness of a fresh agri-food supply chain to disruptions

**DOI:** 10.1007/s12351-023-00769-7

**Published:** 2023-04-08

**Authors:** Ana Esteso, M. M. E. Alemany, Fernando Ottati, Ángel Ortiz

**Affiliations:** 1 Research Centre on Production Management and Engineering (CIGIP), Universitat Politècnica de València, Camino de Vera S/N, 46022 Valencia, Spain; 2 Universitat Politècnica de València, Camino de Vera S/N, 46022 Valencia, Spain; 3 Palo Alto, c/Jesús Dávila y Cornelio Merchán Edif, La Quinta Ofc. 6, 010204 Cuenca, Ecuador

**Keywords:** Agri-food supply chain, Disruption, Robustness, Simulation, System dynamics

## Abstract

The agri-food sector is subject to various sources of uncertainty and risk that can have a negative impact on its supply chain performance if not properly managed. In order to determine what actions the supply chain (SC) should take to protect itself against risks, it is necessary to analyze whether the supply chain is robust to them. This paper proposes a tool based on a system dynamics model to determine the robustness of an already designed five-stage fresh agri-food supply chain (AFSC) and its planting planning to disruptions in demand, supply, transport, and the operability of its nodes. The model is validated using the known behavior replication test and the extreme conditions test. In order to guide decision-makers in the different uses of the above system dynamic model, a methodology for the improvement of the AFSC robustness is presented and applied to a case study. As a result, the SC robustness to the defined disruptions is provided. For critical disruptions, protective actions are defined. Finally, the model is re-run to evaluate the impact of these proactive strategies on the AFSC in order to finally select the most beneficial for improving its robustness.

## Introduction

Agri-food supply chains (AFSCs) are subject to numerous sources of uncertainty and risk that can have a negative impact on them if not properly managed (Esteso et al. 2017). Risk refers to imperfect knowledge when the probability of possible outcomes is known, while uncertainty exists when these probabilities are not known (Hardaker et al. 2015). In the supply chain (SC) context, risk is the undesirable deviation from expected outcomes that may adversely affect SC operations (Tummala and Schoenherr 2011).

As the agri-food sector contributes to the European economy with 4.25 million employees and a turnover of more than a billion of euros (Esteso et al. 2018), risks need to be properly managed to have the least negative impact on their SCs. AFSCs are subject to disruption risks, which are unexpected events arising from events caused by natural or human factors that involve changes in the structure of the SC (Gaonkar and Viswanadham 2007). The most relevant risks in AFSCs are those related to the demand and supply of agri-food products, the inoperability of nodes, and the impossibility of transporting vegetables between two nodes of the SC.

Disruptions in demand may be caused for example by changes in consumers’ eating habits and panic buying during a pandemic (Jámbor et al. 2020), among others. Disruptions in supply could be related, for example, to changes in crop yields or to fire or flooding on farms (Coluccia et al. 2021). Disruptions in node operability can be caused by the closure of SC nodes due to natural disasters such as floods or fires, labor outbreaks (Hobbs 2021), among others. Transport disruptions can emerge in SCs with nodes in different regions or countries between which passage is banned (e.g. in an attempt to contain the spread of a virus like COVID-19 (European Parliament 2020)), or due to inclement weather among others.

The ability of a SC to withstand the disruptive events to which it is subject is called robustness (Pettersen and Asbjørnslett 2019). Knowing the degree of robustness (or lack thereof) of the AFSC to certain disruptions provides SC members with relevant information to be able to take protective actions against risks. However, there are no known simulation tools in the literature to analyze the robustness of AFSCs to disruptions in demand, supply, transport, and SC node operability.

To fill this gap, this paper aims is to design a tool to analyze, measure and improve the robustness of a fresh AFSC configuration and its planting planning to all the above disruptions. For this purpose, a system dynamics (SD) model is developed that represents the management of a complete AFSC consisting of farms, packing plants (PPs), warehouses, distribution centers (DCs) and markets, in which decisions related to the cultivation, harvesting, packing, transport, storage, handling, waste and sales of fresh vegetables, and the hiring and firing of permanent and temporary workers are addressed. The SD model can be applied for different purposes: (a) to support the operation of the AFSC in an “as usual” scenario with no disruptions (named as baseline scenario) by establishing the value of all above decisions, (b) to assess the robustness of the AFSC under different disruptions and/or combination of them, (c) to evaluate possible protective actions on the AFSC robustness, including its redesign and (d) to select the most properly protective action as a course of action.

Since multiple uses of the AFSC SD model are possible, a methodology to assist managers in boosting the AFSC robustness is also proposed. Once the model is validated, the methodology is applied to study the robustness of an AFSC to disruptions through “what-if” scenarios. After analyzing the scenarios for which the AFSC is not robust, protective actions can be taken to increase its robustness, and re-run the model to check whether the actions taken have had the expected effect on the robustness of the SC. Therefore, the contribution of this research is manifold:

- A novel SD model, including inherent characteristics of agri-food sector not previously modelled or not simultaneously with others, is defined that can be used for supporting the whole AFSC planning under normal operation conditions.
- A novel SD model that can be employed to measure the robustness of the AFSC in a proactive or reactive manner under different disruptions not jointly considered in other simulation models. These disruptions affect not only the AFSC operation but also its design.
- A methodology to support managers in the assessment, analysis and improvement of the AFSC robustness based on the different uses of the SD model.

The rest of the paper is structured as follows. Section 2 reviews simulation models for managing AFSCs and identifies the main contributions of the paper. Section 3 describes the problem under study. Section 4 formulates the causal diagram and the flow diagram representing the problem and validates the simulation model. Section 5 presents the methodology to support managers in the use of the SD model to assess and enhance the robustness of AFSCs meanwhile its application to a case study is made in Sect. 6. Finally, Sect. 7 outlines the main conclusions and future research lines.

## Related literature analysis and contributions of this study

This section does not aim to establish the state of the art in this area, but rather to analyze the characteristics of simulation models for managing AFSCs that are relevant to this work, and to identify their main differences with the proposal in this paper to show their originality. The identified models are analyzed in terms of the modelling tool used, the SC stages included, the disruptions studied, the decisions made by the models, and the product characteristics considered (Tables 1 and 2).

**Table 1:** Characteristics of simulation models for AFSC management (Part 1)

Author/s	Modelling tool					SC configuration					Disruption modelling						
	SS	DE	AB	SD	O	F	Pr	W	DC	M	D	S	T	NO	Ca	Co	N
Briano et al. (2010)				X			X			X							X
Aiello et al. (2012)	X					X		X		X							X
Meng et al. (2012)		X				X											X
Ge et al. (2015)			X			X	X										X
Huff et al. (2015)				X		X	X		X	X	X						
Leblanc et al. (2015)		X				X	X		X	X							X
Schätter et al. (2015)					X					X		X	X				
Ge et al. (2016)					X	X	X										X
Liu et al. (2016)				X	X	X			X	X							X
Marchand et al. (2016)	X					X			X	X		X					
Hasani et al. (2018)					X	X	X		X	X							X
Rozhkov and Ivanov (2018)		X	X				X		X						X		
Granillo-Macias et al. (2019)		X				X	X										X
Mahfouz et al. (2019)		X	X						X	X	X						
Verwaart et al. (2019)			X			X				X							X
Clark et al. (2020)				X			X		X	X							X
Ivanov and Rozhkov (2020)		X	X						X	X					X		
Namany et al. (2020)			X			X			X			X					
Singh et al. (2020)								X	X	X			X	X			
This paper				X		X	X	X	X	X	X	X	X	X			

SS: spreadsheet-based simulation, DES: discrete event simulation, AB: agent-based simulation, SD: system dynamics, O: other; F: farm, Pr: processor, W: warehouse, DC: distribution center, M: market; D: demand, S: supply, T: transport, NO: node operability, Ca: capacity, Co: contamination, N: None

**Table 2:** Characteristics of simulation models for AFSC management (Part 2)

Author/s	Decisions													Product characteristics	
	FL	MA	C	H	P	Ha	Inv	O	T	W	UD	L	Pr	Pe	Q
Briano et al. (2010)							X				X		X	X	
Aiello et al. (2012)				X			X		X					X	
Meng et al. (2012)			X												
Ge et al. (2015)									X						
Huff et al. (2015)							X		X					X	
Leblanc et al. (2015)					X			X	X						
Schätter et al. (2015)	X	X										X			
Ge et al. (2016)									X						
Liu et al. (2016)							X	X							
Marchand et al. (2016)								X					X		
Hasani et al. (2018)							X	X						X	
Rozhkov and Ivanov (2018)								X	X				X	X	
Granillo-Macias et al. (2019)	X								X						
Mahfouz et al. (2019)									X					X	X
Verwaart et al. (2019)				X					X						
Clark et al. (2020)									X						
Ivanov and Rozhkov (2020)							X	X	X	X				X	
Namany et al. (2020)				X			X						X		
Singh et al. (2020)									X						
This paper			X	X	X	X	X		X	X	X	X		X	

FL: facility location, MA: market allocation, C: cultivation, H: harvesting, P: packing, Ha: handling, Inv: inventory, O: order, T: transport, W: waste, UD: unsatisfied demand, L: labor, Pr: procurement; Pe: perishability, Q: quality

The simulation tools most used in the papers reviewed are agent-based and discrete event simulation models (Table 1). System dynamics has mainly been used to address strategic problems (e.g. analyzing the impact of pandemic-related absenteeism on food systems (Huff et al. 2015)), being this paper the first to use it to address also tactical problems such as planning the production and distribution of vegetables through the AFSC. Spreadsheet-based simulation has been less used and to solve simple problems.

None of the models analyzed have considered all stages of the AFSC (farms, processors, warehouses, DCs and markets). This is because most of them address strategic problems and therefore focus on only one part of the AFSC, such as the agricultural part (farm) or the marketing part (DCs and markets). This paper fills this gap in the literature by addressing tactical problems such as the planning of the production and distribution of vegetables along a five-stage AFSC.

Only 42% of the models analyzed include some disruption in the AFSC, such as disruptions in demand, supply, transport, SC node operability, facilities capacity, or contamination. Up to our knowledge, this paper is the first to analyze the robustness of the AFSC to disruptions in demand, supply, transport, and SC node operability.

Most of models (Table 2) focus on simulating specific problems such as vegetable transport or inventory planning that allow the analysis of possible policies to be adopted by AFSC members in various scenarios. Therefore, none of the research analyzed models the decisions taken by the AFSC to ensure the flow of vegetables from farms to markets such as harvesting, packing, storage, and handling of vegetables, or the use of transport and labor, and this paper is the first to propose a model considering all these decisions.

Regarding the characteristics of the agri-food products, only 37% of the models model the perishability of the products and 5% model the quality of the products by linking it to the shelf-life of the product, thus being an indicator of their perishability. Only four papers (Huff et al 2015; Rozhkov and Ivanov 2018; Mahfouz et al. 2019; Ivanov and Rozhkov 2020) consider the perishability jointly with one disruption, demand, or capacity, but anyone with more than one disruption at the same time, as our SD model.

Therefore, this paper offers multiple novelties to the literature, which are: i) use of system dynamics to address tactical-operative decisions in agri-food sector, ii) simulation of the management of a complete fresh AFSC consisting of farms, PPs, warehouses, DCs, and markets, iii) joint modelling of the tactical-operational decisions of harvesting, packing, storing, handling, transporting, selling, and wasting vegetables, usage of trucks and hiring/firing of permanent and temporary workers at the farm level, iv) analysis of the robustness of an AFSC configuration and its planting planning to disruptions in demand, supply, transport between SC nodes, and SC nodes operability simultaneously and jointly with the shelf-life and v) implementing and assessing the impact of preventive actions including the redesign of the AFSC.

## Problem description

**Fig. 1:**
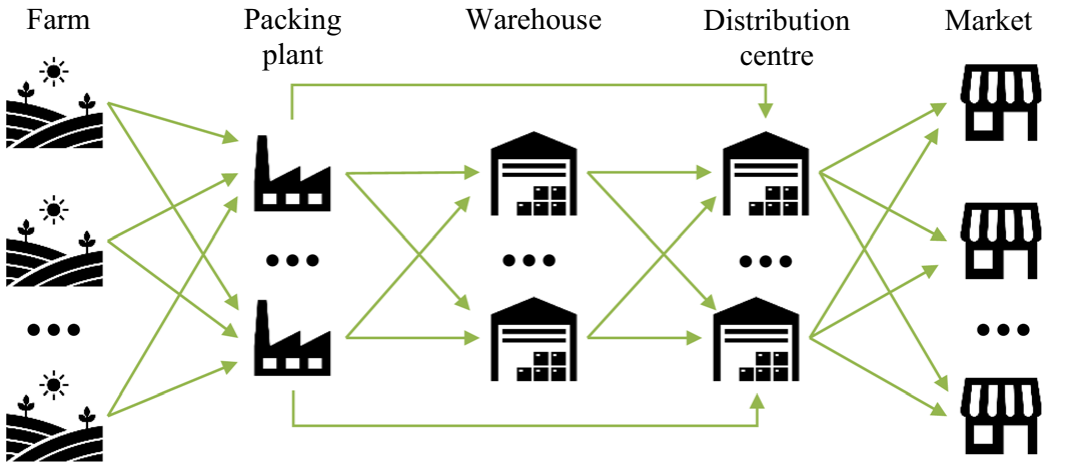
Agri-food SC configuration

This paper aims to determine the robustness of an already designed AFSC for perishable vegetables and its plantation planning to disruptions in demand, supply, transport, and the operability of the SC nodes. The AFSC under study consists of farms, packing plants (PPs), warehouses, distribution centers (DCs) and markets (Fig. 1), and it deals with the cultivation and harvest of planted areas and the packing and distribution of several perishable vegetables.

The proposed simulation model to determine the robustness of the AFSC configuration and its planting planning works under the following assumptions:

- It simulates the flow of vegetables through the AFSC, defining the quantity of vegetables to be harvested, packed, transported, stored, wasted, and sold. It also defines the resources required for this in terms of usage of trucks and hiring/firing of permanent and temporary workers at the farm level.
- The configuration of the SC is defined so it is known which nodes have been selected at each stage to form the SC. The nodes likely to form part of the SC are known. It is possible to model the disruption of shut-down some open nodes and to select new ones in the event of a disruption. Therefore, the SD model allows the redesign of the AFSC in the event of a disruption by opening/closing some of its nodes.
- Farms are responsible for planting, cultivating, and harvesting vegetables. On each farm, planting of specific plants has been carried out on specific planting dates. The area planted with each plant in each planting period is known.
- The planting and harvesting schedule for each plant and the yield of the plants according to this schedule is known. Plants are cultivated from the time the seed is planted until the last period when the plant is harvested (Alemany et al. 2021).
- Planting, cultivation and harvesting of vegetables are carried out manually and the time required for these activities depends on the vegetable and is known.
- Farms can hire and fire temporary or permanent workers to perform the manual activities of planting, cultivating, and harvesting vegetables. Farms have a minimum number of permanent workers dependent on the farm area that cannot be dismissed.
- The number of temporary and permanent workers available for hire on all farms is limited. Temporary and permanent workers share the same working hours. The hiring of permanent workers has priority over the hiring of temporary workers.
- Once the vegetables are harvested, they are immediately transported to the PPs to avoid deterioration. In the PPs vegetables can be stored prior to packing or packed. The storage and packing capacity are limited and known.
- After packing, the vegetables are loaded onto trucks for transport to warehouses or DCs. Vegetables are transported from warehouses to DCs. Handling and storage capacity at warehouses and DCs are limited and known. The storage capacity of DCs is much more limited than that of warehouses, as their main function is to consolidate vegetables received for distribution, and not for long-term storage.
- Vegetables are transported from the DCs to the markets, where the demand for vegetables is met. If there are not enough vegetables available to meet the demand, an unsatisfied demand is generated.
- Once harvested, vegetables have a limited shelf-life that decreases over time. Vegetables are wasted when their shelf-life is shorter than required by customers. This waste can be generated in PPs, warehouses, DCs and markets.
- Trucks are used to transport vegetables between nodes in the SC, which have limited transport capacity. The time required to transport the vegetables between the different nodes of the SC is known. Vegetables are transported to the closest node in the following SC stage.
- The price of vegetables and the costs of planting and cultivating (seeds, water, phytosanitary products, etc.), packing, storing, and handling of vegetables, hiring, firing and wages of permanent and temporary workers, use of SC nodes, use of trucks, and the economic penalty for unmet demand and waste generation are known.

Furthermore, this paper considers that the AFSC may be subject to disruptions in demand, supply, transport, and SC node operation. To test the robustness of the SC to these disruptions, the proposed model will be run for several “what-if” scenarios.

## Formulation and validation of the system dynamics model

Following the assumptions made and based on the mathematical programming model for the design and management of the AFSC for perishable vegetables proposed in Esteso et al. (2021), the system dynamics model is proposed.

A five-phase methodology based on Esteso et al. (2019) is followed: (i) propose the casual-loop diagram, (ii) create the flow diagram representing the SC and the processes required to manage it, (iii) generate the equations defining the behavior of the system dynamics model, (iv) validate the system dynamics model by comparing it with the results obtained by the equivalent optimization model, and (v) evaluate the results obtained for several “what-if” scenarios representing disruptions in the AFSC.

### Causal-loop diagram

**Fig. 2:**
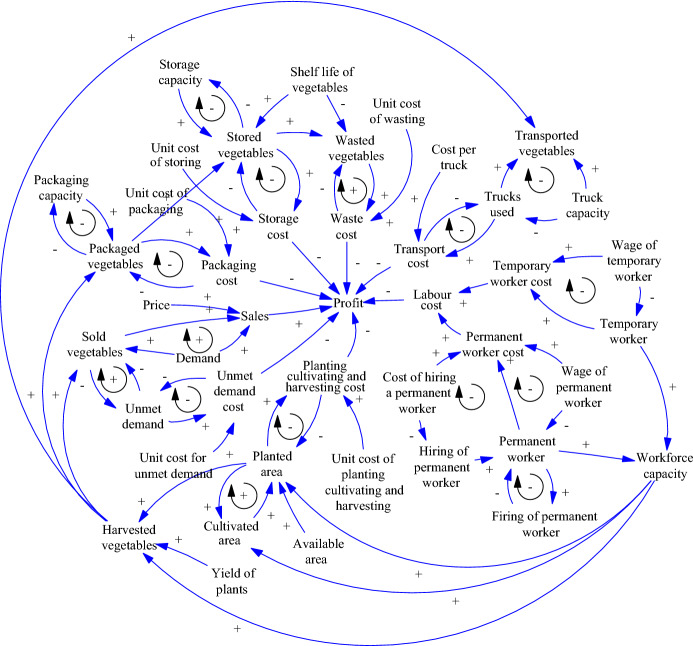
Causal-loop diagram of AFSC management

The casual-loop diagram (Fig. 2) shows the cause-effect relations between the elements of the AFSC, which helps to understand its operation and subsequent modelling in the flow diagram. These relations are represented by arrows that include a positive sign when two variables are directly proportional (the increase of one variable causes the increase of the other) and a negative sign when two variables are inversely proportional (the increase of one variable causes the decrease of the other).

The loops formed by the relations of the system elements are negative when they act as stabilizers of the system as they lead to a particular goal, and positive when they have the opposite effect on the system (Esteso et al. 2019). In this case, the modelled system is hyperstable as most of the loops are negative.

### Flow diagram

The nomenclature used to define the level, flow and auxiliary variables used in the formulation of the system dynamics model is shown in Table 3, where the index v refers to the vegetable, f to the farm, c to the PP, w to the warehouse, d to the DC, m to the market, p to the planting period and h to the harvesting period.

**Table 3:** Level, flow, and auxiliary variables

**Level variables**	
${TAP}_{vf}^{p}$	Total area planted with vegetable *v* on farm *f* in planting period *p* (ha)
${TAC}_{vf}^{p}$	Total cultivated area of vegetable *v* on farm *f* in planting period *p* (ha)
${TAH}_{vf}^{ph}$	Total harvested area of vegetable *v* planted in planting period *p* on farm *f* in harvest period *h* (ha)
${TLR}_{vf}^{p}$	Total labor requirement for planting, cultivation and harvesting of vegetable *v* planted in planting period *p* on farm *f* (min)
${TPL}_{f}$	Total amount of permanent labor used on farm *f* (worker)
${TTL}_{f}$	Total amount of temporary labor used on farm *f* (worker)
${TQH}_{vf}^{h}$	Total quantity of vegetable *v* harvested on farm *f* in harvest period *h* (kg)
${TIP}_{vc}^{h}$	Total inventory of vegetable *v* harvested in period *h* stored in PP *c* (kg)
${TQP}_{vc}^{h}$	Total quantity of vegetable *v* harvested in period *h* packed at PP *c* (kg)
${TIW}_{vw}^{h}$	Total inventory of vegetable *v* harvested in period *h* stored in the warehouse *w* (kg)
${TID}_{vd}^{h}$	Total inventory of vegetable *v* harvested in period *h* stored in the DC *d* (kg)
${TIM}_{vm}^{h}$	Total inventory of vegetable *v* harvested in period *h* stored in the market *m* (kg)
${TWP}_{vc}^{h}$	Total quantity of vegetable *v* harvested in period *h* wasted in the PP *c* (kg)
${TWW}_{vw}^{h}$	Total quantity of vegetable *v* harvested in period *h* wasted in the warehouse *w* (kg)
${TWD}_{vd}^{h}$	Total quantity of vegetable *v* harvested in period *h* wasted in the DC *d* (kg)
${TWM}_{vm}^{h}$	Total quantity of vegetable *v* harvested in period *h* wasted in the market *m* (kg)
${TTFP}_{fc}$	Total number of trucks used to transport vegetables from farm *f* to PP *c* (truck)
${TTPW}_{cw}$	Total number of trucks used to transport vegetables from PP *c* to warehouse *w* (truck)
${TTPD}_{cd}$	Total number of trucks used to transport vegetables from PP *c* to DC *d* (truck)
${TTWD}_{wd}$	Total number of trucks used to transport vegetables from warehouse *w* to DC *d* (truck)
${TTDM}_{dm}$	Total number of trucks used to transport vegetables from DC *d* to market *m* (truck)
${TD}_{vm}$	Total demand for vegetable *v* at market *m* (kg)
${TSD}_{vm}$	Total satisfied demand for vegetable *v* at market *m* (kg)
${TUD}_{vm}$	Total unsatisfied demand for vegetable *v* at market *m* (kg)
$Profit$	Total profit of the AFSC (€)
**Flow variables**	
${AP}_{vf}^{p}$	Area planted with vegetable *v* by farm *f* in the planting period *p* (ha/week)
${AC}_{vf}^{p}$	Area planted in planting period *p* with vegetable *v* on farm *f* to be cultivated (ha/week)
${AH}_{vf}^{ph}$	Area planted in planting period *p* with vegetable *v* on farm *f* to be harvested in period *h* (ha/week)
${TP}_{vf}^{p}$	Time needed to plant the vegetable *v* in the planting period *p* on farm *f* (min/week)
${TC}_{vf}^{p}$	Time needed to cultivate the area planted in period *p* with vegetable *v* on farm *f* (min/week)
${TH}_{vf}^{ph}$	Time needed to harvest the area planted in period *p* with vegetable *v* on farm *f* in harvest period *h* (min/week)
${HPL}_{f}$	Number of permanent laborers hired on farm *f* (worker/week)
${FPL}_{f}$	Number of permanent laborers fired on farm *f* (worker/week)
${HTL}_{f}$	Number of temporary laborers hired on farm *f* (worker/week)
${FTL}_{f}$	Number of temporary laborers fired on farm *f* (worker/week)
${QH}_{vf}^{ph}$	Quantity of vegetable *v* planted in period *p* on farm *f* harvested in harvest period *h* (kg/week)
${QFP0}_{vfc}^{h}$	Quantity of vegetable *v* harvested in harvest period *h* transported from farm *f* to PP *c*, where transport time is zero (kg/week)
${QFP1}_{vfc}^{h}$	Quantity of vegetable *v* harvested in harvest period *h* transported from farm *f* to PP *c*, where transport time is greater than zero (kg/week)
${DFP}_{vfc}^{h}$	Dummy variable used to retain the vegetables *v* harvested in harvest period *h* between farm *f* and PP *c* for the time equivalent to the transport time between them (kg/week)
${QP}_{vc}^{h}$	Quantity of vegetable *v* harvested in harvest period *h* packed in the PP *c* (kg/week)
${QPW0}_{vcw}^{h}$	Quantity of vegetable *v* harvested in harvest period *h* transported from PP *c* to warehouse *w*, where transport time is zero (kg/week)
${QPW1}_{vcw}^{h}$	Quantity of vegetable *v* harvested in harvest period *h* transported from PP *c* to warehouse *w*, where transport time is greater than zero (kg/week)
${DPW}_{vcw}^{h}$	Dummy variable used to retain the vegetables *v* harvested in harvest period *h* between PP *c* and warehouse *w* for the time equivalent to the transport time between them (kg/week)
${QPD0}_{vcd}^{h}$	Quantity of vegetable *v* harvested in harvest period *h* transported from PP *c* to DC *d*, where transport time is zero (kg/week)
${QPD1}_{vcd}^{h}$	Quantity of vegetable *v* harvested in harvest period *h* transported from PP *c* to DC *d*, where transport time is greater than zero (kg/week)
${DFD}_{vcd}^{h}$	Dummy variable used to retain the vegetables *v* harvested in harvest period *h* between PP *c* and DC *d* for the time equivalent to the transport time between them (kg/week)
${QWD0}_{vwd}^{h}$	Quantity of vegetable *v* harvested in harvest period *h* transported from warehouse *w* to DC *d*, where transport time is zero (kg/week)
${QWD1}_{vwd}^{h}$	Quantity of vegetable *v* harvested in harvest period *h* transported from warehouse *w* to DC *d*, where transport time is greater than zero (kg/week)
${DWD}_{vwd}^{h}$	Dummy variable used to retain the vegetables *v* harvested in harvest period *h* between warehouse *w* and DC *d* for the time equivalent to the transport time between them (kg/week)
${QDM0}_{vdm}^{h}$	Quantity of vegetable *v* harvested in harvest period *h* transported from DC *d* to market *m*, where transport time is zero (kg/week)
${QDM1}_{vdm}^{h}$	Quantity of vegetable *v* harvested in harvest period *h* transported from DC *d* to market *m*, where transport time is greater than zero (kg/week)
${DDM}_{vdm}^{h}$	Dummy variable used to retain the vegetables *v* harvested in harvest period *h* between DC *d* and market *m* for the time equivalent to the transport time between them (kg/week)
${NFP}_{fc}$	Number of trucks used to transport vegetables from farm *f* to PP *c* (truck/week)
${NPW}_{cw}$	Number of trucks used to transport vegetables from PP *c* to warehouse *w* (truck/week)
${NPD}_{cd}$	Number of trucks used to transport vegetables from PP *c* to DC *d* (truck/week)
${NWD}_{wd}$	Number of trucks used to transport vegetables from warehouse *w* to DC *d* (truck/week)
${NDM}_{dm}$	Number of trucks used to transport vegetables from DC *d* to market *m* (truck/week)
${WSP}_{vc}^{h}$	Quantity of vegetable *v* harvested in harvest period *h* wasted at PP *c* when its shelf-life is less than the required by the customer (kg/week)
${WCP}_{vc}^{h}$	Quantity of vegetable *v* harvested in harvest period *h* wasted at PP *c* due to exceeding its storage and packing capacity (kg/week)
${WSW}_{vw}^{h}$	Quantity of vegetable *v* harvested in harvest period *h* wasted at warehouse *w* when its shelf-life is less than the required by the customer (kg/week)
${WCW}_{vw}^{h}$	Quantity of vegetable *v* harvested in harvest period *h* wasted at warehouse *w* due to exceeding its storage and handling capacity (kg/week)
${WSD}_{vd}^{h}$	Quantity of vegetable *v* harvested in harvest period *h* wasted at DC *d* when its shelf-life is less than the required by the customer (kg/week)
${WCD}_{vd}^{h}$	Quantity of vegetable *v* harvested in harvest period *h* wasted at DC *d* due to exceeding its storage and handling capacity (kg/week)
${WSM}_{vm}^{h}$	Quantity of vegetable *v* harvested in harvest period *h* wasted at market *m* when its shelf-life is less than the required by the customer (kg/week)
${D}_{vm}$	Demand for vegetable *v* at the market *m* (kg/week)
${QS}_{vm}^{h}$	Quantity of vegetable *v* harvested in harvest period *h* sold on the market *m* (kg/week)
${SD}_{vm}$	Quantity of satisfied demand for vegetable *v* on the market *m* (kg/week)
${UD}_{vm}$	Quantity of unsatisfied demand for vegetable *v* on the market *m* (kg/week)
$Inc$	Income from the sale of vegetables on the markets (€/week)
$CostPCH$	Cost of planting, cultivation, and harvesting of vegetables planted by farms (€/week)
$CostHPL$	Cost of hiring permanent laborers (€/week)
$CostPL$	Cost of wages for permanent laborers (€/week)
$CostTL$	Cost of wages for temporary laborers (€/week)
$CostP$	Cost of packing vegetables (€/week)
$CostFP$	Cost of transporting vegetables from farms to PPs (€/week)
$CostPW$	Cost of transporting vegetables from PPs to warehouses (€/week)
$CostPD$	Cost of transporting vegetables from PPs to DCs (€/week)
$CostTWD$	Cost of transporting vegetables from warehouses to DCs (€/week)
$CostDM$	Cost of transporting vegetables from DCs to markets (€/week)
$CostIP$	Cost of storage of vegetables in PPs (€/week)
$CostIW$	Cost of storage of vegetables in warehouses (€/week)
$CostID$	Cost of storage of vegetables in DCs (€/week)
$CostHW$	Cost of handling vegetables in warehouses (€/week)
$CostHD$	Cost of handling vegetable in DCs (€/week)
$CostWP$	Cost of wastage of vegetables in PPs (€/week)
$CostWW$	Cost of wastage of vegetables in warehouses (€/week)
$CostWD$	Cost of wastage of vegetables in DCs (€/week)
$CostWM$	Cost of wastage of vegetables in markets (€/week)
$CostUD$	Cost of not meeting market demand (€/week)
**Auxiliary variables**	
${a}_{vf}^{p}$	Area planted with vegetable *v* in planting period *p* on farm *f* (hectare)
${tplant}_{v}$	Period in which vegetable *v* can be planted (week)
${th}_{v}$	Period in which vegetable *v* can be harvested (week)
${pc}_{v}^{p}$	Binary variable taking value of one if the vegetable *v* planted in planting period *p* needs to be cultivated, otherwise zero
${ph}_{v}^{ph}$	Binary variable taking value of one if the vegetable *v* planted in planting period *p* needs to be harvested in harvesting period h, otherwise zero
${timp}_{v}$	Time needed to plant one hectare of vegetable *v* (min/hectare)
${timc}_{v}$	Time needed to cultivate one hectare of vegetable *v* (min/hectare)
${timh}_{v}$	Time needed to harvest one hectare of vegetable *v* (min/hectare)
${y}_{v}^{ph}$	Quantity of vegetable *v* obtained per plant if planted in period *p* and harvested in period *h* (kg/plant)
${dem}_{vm}$	Quantity of vegetable *v* demanded by market *m* (kg)
$nm$	Number of markets (market)
$sl$	Shelf-life of vegetables (week)
$msl$	Minimum shelf-life of vegetables sold due to freshness and quality requirements (week)
${sp}_{c}$	Vegetable storage capacity of PP *c* (kg)
${sw}_{w}$	Vegetable storage capacity of warehouse *w* (kg)
${sid}_{d}$	Vegetable storage capacity of DC *d* (kg)
${pp}_{c}$	Vegetable packing capacity of PP *c* (kg)
${hw}_{w}$	Vegetable handling capacity of warehouse *w* (kg)
${hd}_{d}$	Vegetable handling capacity of DC *d* (kg)
${tfp}_{fc}$	Transport time of vegetables from farm *f* to PP *c* (week)
${tpw}_{cw}$	Transport time of vegetables from PP *c* to warehouse *w* (week)
${tpd}_{cd}$	Transport time of vegetables from PP *c* to DC *d* (week)
${tiwd}_{wd}$	Transport time of vegetables from warehouse *w* to DC *d* (week)
${tdm}_{dm}$	Transport time of vegetables from DC *d* to market *m* (week)
$mtl$	Maximum truck load (kg/truck)
$wt$	Working time of laborers (min/week)
$maxp$	Maximum number of permanent laborers (worker)
$maxt$	Maximum number of temporary laborers (worker)
${minp}_{f}$	Minimum permanent labor force on farm *f* (worker)
${p}_{vm}$	Unit selling price of vegetable *v* on the market *m* (€/kg)
$cpch$	Unit cost of planting, cultivation, and harvesting of vegetable *v* (€/hectare)
$chpl$	Cost of hiring a permanent laborer (€/worker)
$cpl$	Weekly wage of a permanent laborer (€/worker)
$ctl$	Weekly wage of a temporary laborer (€/worker)
${cp}_{v}$	Unit cost of packing vegetable *v* at packing plants (€/kg)
$cf{p}_{fp}$	Unit cost of transporting a truck from farm *f* to PP *c* (€/truck)
$cp{w}_{cw}$	Unit cost of transporting a truck from PP *c* to warehouse *w* (€/truck)
$cp{d}_{cd}$	Unit cost of transporting a truck from PP *c* to DC *d* (€/truck)
$ctw{d}_{wd}$	Unit cost of transporting a truck from warehouse *w* to DC *d* (€/truck)
$cd{m}_{dm}$	Unit cost of transporting a truck from DC *d* to market *m* (€/truck)
${cip}_{v}$	Unit cost for storing vegetable *v* in the PPs per period (€/kg·week)
${ciw}_{v}$	Unit cost for storing vegetable *v* in the warehouses per period (€/kg·week)
${cid}_{v}$	Unit cost for storing vegetable *v* in the DCs per period (€/kg·week)
${chw}_{v}$	Unit cost for handling the vegetable *v* in the warehouses (€/kg)
${chd}_{v}$	Unit cost for handling the vegetable *v* in the DCs (€/kg)
${cwp}_{v}$	Unit cost for vegetable *v* wastage in PPs (€/kg)
${cww}_{v}$	Unit cost for vegetable *v* wastage in warehouses (€/kg)
${cwd}_{v}$	Unit cost for vegetable *v* wastage in DCs (€/kg)
${cwm}_{v}$	Unit cost for vegetable *v* wastage in markets (€/kg)
${cud}_{vm}$	Unit cost of not meeting market *m* demand for vegetable *v* (€/kg)

**Fig. 3:**
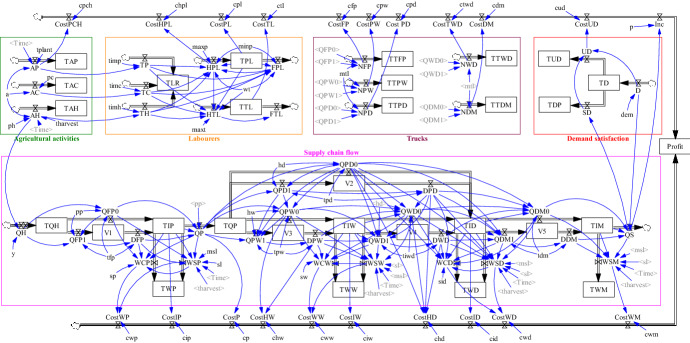
Flow diagram of agri-food supply chain management

Figure 3 shows the flow diagram representing the management of the AFSC under study, which allows analyzing the robustness of the AFSC under various disruptions trough “what if” scenarios. For this purpose, the model is implemented in the simulation software Vensim® DSS for Windows Version 6.3G.

### Definition of equations

In this section, the equations that regulate the operation of previously defined level and flow variables are defined. As for the auxiliary variables, which introduce the data into the model, they import their values from a spreadsheet created in Microsoft Excel.

The simulation starts by assigning to the flow variable ${AP}_{vf}^{p}$ the value of the area to be planted with each vegetable on each farm in each planting period (1). This is done by assigning the value of the auxiliary variable ${a}_{vf}^{p}$ in the corresponding planting periods.

1 $${AP}_{vf}^{p}\left(t\right)=\left\{\begin{array}{l}if\quad {tplant}_{v}\left(t\right)=t: \left[{a}_{vf}^{p}\right] \\ otherwise: \left[0\right] \end{array}\right.\quad \forall\; v,f,p$$

The total planted area ${TAP}_{vf}^{p}$ is the accumulation of the planted areas for each vegetable, farm, and planting period, being its value at the beginning of the simulation zero (2).

2 $${TAP}_{vf}^{p}\left(t\right)=\underset{0}{\overset{t}{\int }}\left({AP}_{vf}^{p}\right) dt;\quad {TAP}_{vf}^{p}\left({t}_{0}\right)=0\quad \forall \; v,f,p$$

The area to be cultivated in each period depends on the cultivation calendar of each vegetable according to its planting period. Thus, in case it is necessary to cultivate a vegetable in one period $\left({pc}_{v}^{p}=1\right)$, the whole planted area is cultivated (3).

3 $${AC}_{vf}^{p}\left(t\right)=\left\{\begin{array}{l}if\quad {pc}_{v}^{p}\left(t\right)=1: \left[{a}_{vf}^{p}\right] \\ otherwise: \left[0\right] \end{array}\right.\quad \forall\; v,f,p$$

The total cultivated area ${TAC}_{vf}^{p}$ is the accumulation of the cultivated areas in all simulation periods, with a value of zero at the beginning of the simulation zero (4).

4 $${TAC}_{vf}^{p}\left(t\right)=\underset{0}{\overset{t}{\int }}\left({AC}_{vf}^{p}\right) dt;\quad {TAC}_{vf}^{p}\left({t}_{0}\right)=0\quad \forall\; v,f,p$$

The area to be harvested in each period depends on the area planted with each vegetable and its planting and harvesting calendar (5). Thus, in the periods corresponding to a harvest period, it is checked whether the period is included in the harvest calendar of the vegetable depending on its planting period. If it is $\left({ph}_{v}^{ph}=1\right)$, the entire planted area is harvested.

5 $${AH}_{vf}^{ph}\left(t\right)=\left\{\begin{array}{l}if\quad {th}_{v}=t: \left\{\begin{array}{l}if\quad {ph}_{v}^{ph}\left(t\right)=1: \left[{a}_{vf}^{p}\right] \\ otherwise: \left[0\right] \end{array}\right. \\ otherwise: \left[0\right] \end{array}\right. \quad\forall \; v,f,p,h$$

The total area to be harvested ${TAH}_{vf}^{ph}$ is the accumulation of the areas harvested in all simulation periods, being its value zero at the beginning of the simulation (6).

6 $${TAH}_{vf}^{ph}\left(t\right)=\underset{0}{\overset{t}{\int }}\left({AH}_{vf}^{ph}\right) dt;\quad {TAH}_{vf}^{ph}\left({t}_{0}\right)=0\quad \forall\; v,f,p,h$$

The time needed to plant vegetables ${TP}_{vf}^{p}$ in each period depends on the area to be planted per period and the time required to plant one hectare of the vegetable (7).

7 $${TP}_{vf}^{p}\left(t\right)={AP}_{vf}^{p}\left(t\right)\cdot {timp}_{v}\quad \forall \; v,f,p$$

The time needed to cultivate vegetables ${TC}_{vf}^{p}$ depends on the area to be cultivated per period and the time required to cultivate one hectare of the vegetable (8).

8 $${TC}_{vf}^{p}\left(t\right)={AC}_{vf}^{p}\left(t\right)\cdot {timc}_{v}\quad \forall \; v,f,p$$

The time needed to harvest vegetables ${TH}_{vf}^{ph}$ depends on the area to be harvested per period and the time required to harvest one hectare of the vegetable (9).

9 $${TH}_{vf}^{ph}\left(t\right)={AH}_{vf}^{ph}\left(t\right)\cdot {timh}_{v} \quad\forall \; v,f,p,h$$

The total time ${TLR}_{vf}^{p}$ spent on planting, cultivating, and harvesting each vegetable for each farmer during the simulation is the accumulation of the time spent on each of these activities (10). The value of this variable at the beginning of the simulation is zero.

10 $${TLR}_{vf}^{p}\left(t\right)=\underset{0}{\overset{t}{\int }}\left({TP}_{vf}^{p}\left(t\right)+{TC}_{vf}^{p}\left(t\right)+\sum _{h}{TH}_{vf}^{ph}\left(t\right)\right) dt;\quad {TLR}_{vf}^{p}\left({t}_{0}\right)=0\quad \forall \;v,f,p$$

Farms must hire and fire permanent and temporary workers to adjust their capacity to the time requirements for planting, cultivating, and harvesting vegetables. As priority is given to permanent workers, to hire ${HPL}_{f}$ them (11), it is checked whether the labor needed on the farm is larger than the current labor force – if so, it is checked whether it is possible to cover the labor need with the permanent workers still available on the market. If it is, as many permanent workers as necessary are hired, and if it is not, only the permanent workers available on the market are hired. If the need for labor does not exceed the number of permanent workers hired, it is checked if the minimum number of permanent workers in the farm is contracted, hiring the missing quantity if necessary.

In the same way in all periods, it is checked whether it is necessary to fire permanent workers ${FPL}_{f}$ (12). For this purpose, it is checked whether the labour required on the farm is less than the current labour force. If so, all permanent workers are laid off except for the minimum required on the farm if the need for labour is less than this minimum requirement, or the surplus of workers if it is not.

Temporary workers are hired ${HTL}_{f}$ only when the labour need cannot be met by permanent workers (13). In that case, if there is enough temporary labour available to meet the labour need, the required number of workers would be hired. Otherwise, only the available temporary workers would be hired.

11 $${HPL}_{f}\left(t\right)=\left\{\begin{array}{l}if\quad INT\left(\frac{\sum _{v,p}\left({TP}_{vf}^{p}\left(t\right)+{TC}_{vf}^{p}\left(t\right)+\sum _{h}{TH}_{vf}^{ph}\left(t\right)\right)}{wt}\right)\\ >{TPL}_{f}\left(t\right): \left\{\begin{array}{l}\begin{array}{l}if\quad \left(INT\left(\frac{\sum _{v,p}\left({TP}_{vf}^{p}\left(t\right)+{TC}_{vf}^{p}\left(t\right)+\sum _{h}{TH}_{vf}^{ph}\left(t\right)\right)}{wt}\right)-{TPL}_{f}\left(t\right)\right)\\<\left(maxp+\sum _{f}\left({TPL}_{f}\left(t\right)-{FPL}_{f}\left(t\right)\right)-\sum _{{f}^{^{\prime}} < f}{HPL}_{{f}^{^{\prime}}}\left(t\right)\right):\\ \left[INT\left(\frac{\sum _{v,p}\left({TP}_{vf}^{p}\left(t\right)+{TC}_{vf}^{p}\left(t\right)+\sum _{h}{TH}_{vf}^{ph}\left(t\right)\right)}{wt}\right)-{TPL}_{f}\left(t\right)\right]\end{array}\\ otherwise: \left[maxp+\sum _{f}\left({TPL}_{f}\left(t\right)-{FPL}_{f}\left(t\right)\right)-\sum _{{f}^{^{\prime}} < f}{HPL}_{{f}^{^{\prime}}}\left(t\right)\right] \end{array}\right.\\ otherwise: \left\{\begin{array}{l}if\quad {TPL}_{f}\left(t\right) < {minp}_{f}: \left[{minp}_{f}-{TPL}_{f}\left(t\right)\right]\\ otherwise: \left[0\right] \end{array}\right. \end{array}\right.\quad\forall \; f$$

12 $${FPL}_{f}\left(t\right)=\left\{\begin{array}{l}if\quad INT\left(\frac{\sum _{v,p}\left({TP}_{vf}^{p}\left(t\right)+{TC}_{vf}^{p}\left(t\right)+\sum _{h}{TH}_{vf}^{ph}\left(t\right)\right)}{wt}\right)\\<{TPL}_{f}(t): \left\{\begin{array}{l}if\quad INT\left(\frac{\sum _{v,p}\left({TP}_{vf}^{p}\left(t\right)+{TC}_{vf}^{p}\left(t\right)+\sum _{h}{TH}_{vf}^{ph}\left(t\right)\right)}{wt}\right)<{minp}_{f}: \left[{TPL}_{f}(t)-{minp}_{f}\right]\\ otherwise: \left[{TPL}_{f}\left(t\right)- INT\left(\frac{\sum _{v,p}\left({TP}_{vf}^{p}\left(t\right)+{TC}_{vf}^{p}\left(t\right)+\sum _{h}{TH}_{vf}^{ph}\left(t\right)\right)}{wt}\right)\right] \end{array}\right.\\ otherwise: \left[0\right] \end{array}\right. \quad \forall \; f$$

13 $${HTL}_{f}\left(t\right)=\left\{\begin{array}{l}if\quad INT\left(\frac{\sum _{v,p}\left({TP}_{vf}^{p}\left(t\right)+{TC}_{vf}^{p}\left(t\right)+\sum _{h}{TH}_{vf}^{ph}\left(t\right)\right)}{wt}\right)\\ >\left({TPL}_{f}(t)+{HPL}_{f}(t)\right): \left\{\begin{array}{l}\begin{array}{l}if\quad \left(INT\left(\frac{\sum _{v,p}\left({TP}_{vf}^{p}\left(t\right)+{TC}_{vf}^{p}\left(t\right)+\sum _{h}{TH}_{vf}^{ph}\left(t\right)\right)}{wt}\right)-{TPL}_{f}(t)-{HPL}_{f}(t)\right) \\ < \left(maxt-\sum _{{f}^{^{\prime}} < f}{HTL}_{{f}^{^{\prime}}}(t)\right):\\ \left[INT\left(\frac{\sum _{v,p}\left({TP}_{vf}^{p}\left(t\right)+{TC}_{vf}^{p}\left(t\right)+\sum _{h}{TH}_{vf}^{ph}\left(t\right)\right)}{wt}\right)-{TPL}_{f}(t)-{HPL}_{f}(t)\right]\end{array}\\ otherwise: \left[maxt-\sum _{{f}^{^{\prime}} < f}{HTL}_{{f}^{^{\prime}}}\left(t\right)\right] \end{array}\right.\\ otherwise: \left[0\right] \end{array}\right. \quad\forall \; f$$

Temporary workers only remain employed for a period (${FTL}_{f}$) after which they will be available on the market for further recruitment (14).

14 $${FTL}_{f}\left(t\right)={HTL}_{f}\left(t\right)\quad \forall \; f$$

The total number of permanent workers ${TPL}_{f}$ (15) and temporary workers ${TTL}_{f}$ (16) working at a farm depends on the number of hires and layoffs during the simulation time, being the initial value for these variables equal to zero.

15 $${TPL}_{f}\left(t\right)=\underset{0}{\overset{t}{\int }}\left({HPL}_{f}\left(t\right)-{FPL}_{f}\left(t\right)\right) dt;\quad {TPL}_{f}\left({t}_{0}\right)=0 \quad\forall \; f$$

16 $${TTL}_{f}\left(t\right)=\underset{0}{\overset{t}{\int }}\left({HTL}_{f}\left(t\right)-{FTL}_{f}\left(t\right)\right) dt;\quad {TTL}_{f}\left({t}_{0}\right)=0\quad \forall\; f$$

Regarding the flow of vegetables through the AFSC, it starts when the vegetables are harvested. The quantity of vegetable harvested ${QH}_{vf}^{ph}$ depends on the area to be harvested and the yield of the plants (17).

17 $${QH}_{vf}^{ph}\left(t\right)={AH}_{vf}^{ph}\left(t\right)\cdot {y}_{v}^{ph}\left(t\right) \quad\forall\; v,f,p,h$$

The variable ${TQH}_{vf}^{ph}$ represents the balance between the quantity of harvested vegetables and the quantity of vegetables transported from the farm to the PPs (18). Its value at the beginning of the simulation is zero.

18 $${TQH}_{vf}^{ph}\left(t\right)={\int }_{0}^{t}\left(\sum _{p}{QH}_{vf}^{ph}(t)-\sum _{c}\left({QFP0}_{vfc}^{h}(t)+{QFP1}_{vfc}^{h}(t)\right)\right)dt;\quad {TQH}_{vf}^{h}\left({t}_{0}\right)=0\quad \forall \; v,f,h$$

As it is not possible to inventory vegetables at the farm, this variable should have a value equal to zero in all periods of the simulation. For this purpose, all harvested vegetables must be transported to the PPs, giving priority to those closest to the farm, i.e., those with the shortest transport time. Thus, to define the quantity of vegetables to be transported to the closest PPs ${QFP0}_{vfc}^{h}$, it is first checked whether the transport time is equal to zero. If it is, it is checked whether the PP has sufficient capacity to pack the vegetables available on the farm. If yes, all vegetables are transported to the PP, and if no, the proportional part of each vegetable that the PP is able to pack is transported (19).

19 $${QFP0}_{vfc}^{h}\left(t\right)=\left\{\begin{array}{l}if\quad {tfp}_{fc}(t)=0\left\{\begin{array}{l}\begin{array}{l}if\quad \left({pp}_{c}-\sum_{v,{f}^{^{\prime}} < f}{QFP0}_{v{f}^{^{\prime}}c}^{h}(t)\right)\\\ge \left(\sum_{v,p}{QH}_{vf}^{ph}(t)-\sum_{v,{c}^{^{\prime}} < c}{QFP0}_{vf{c}^{^{\prime}}}^{h}(t)\right) :\\ \left[\sum_{p}{QH}_{vf}^{ph}(t)-\sum _{{c}^{^{\prime}} < c}{QFP0}_{vf{c}^{^{\prime}}}^{h}(t)\right]\end{array}\\ \begin{array}{l}otherwise: \\ \left[\frac{\left(\sum_{p}{QH}_{vf}^{ph}(t)-\sum _{{c}^{^{\prime}} < c}{QFP0}_{vf{c}^{^{\prime}}}^{h}(t)\right)\cdot \left({pp}_{c}-\sum _{v,{f}^{^{\prime}} < f}{QFP0}_{v{f}^{^{\prime}}c}^{h}(t)\right)}{\sum_{v,p}{QH}_{vf}^{ph}(t)-\sum _{v,{c}^{^{\prime}} < c}{QFP0}_{vf{c}^{^{\prime}}}^{h}(t)}\right]\end{array} \end{array}\right.\\ otherwise: \left[0\right] \end{array}\right.\quad \forall \; v,f,c,h$$

Similarly, in the case where, having transported vegetables to the nearest packing plants (${tfp}_{fc}=0$), there were still vegetables left on the farm, these would be sent to the PPs with a transport time greater than zero following the same logic (20).

20 $${QFP1}_{vfc}^{h}\left(t\right)=\left\{\begin{array}{l}if\quad {tfp}_{fc}\left(t\right)>0\left\{\begin{array}{l}\begin{array}{l}\begin{array}{l}if\quad \left({pp}_{c}-\sum _{v,f}{QFP0}_{vfc}^{h}\left(t\right)-\sum _{v,{f}^{^{\prime}}<f}{QFP1}_{v{f}^{^{\prime}}c}^{h}\left(t\right)\right)\ge \\ \left(\sum_{v,p}{QH}_{vf}^{ph}\left(t\right)-\sum _{v,c}{QFP0}_{vfc}^{h}\left(t\right)-\sum _{v,{c}^{^{\prime}}<c}{QFP1}_{vf{c}^{^{\prime}}}^{h}\left(t\right)\right) :\end{array} \\ \left[\sum_{p}{QH}_{vf}^{ph}\left(t\right)-\sum _{c}{QFP0}_{vfc}^{h}\left(t\right)-\sum _{{c}^{^{\prime}}<c}{QFP1}_{vf{c}^{^{\prime}}}^{h}\left(t\right)\right]\end{array}\\ \begin{array}{l}otherwise: \\ \begin{array}{l}\left[\left(\sum_{p}{QH}_{vf}^{ph}\left(t\right)-\sum _{c}{QFP0}_{vfc}^{h}\left(t\right)-\sum _{{c}^{^{\prime}}<c}{QFP1}_{vf{c}^{^{\prime}}}^{h}\left(t\right)\right)\cdot \right.\\ \left.\frac{\left({pp}_{c}-\sum _{v,f}{QFP0}_{vfc}^{h}\left(t\right)-\sum _{v,{f}^{^{\prime}}<f}{QFP1}_{v{f}^{^{\prime}}c}^{h}\left(t\right)\right)}{\sum_{v,p}{QH}_{vf}^{ph}\left(t\right)-\sum _{v,c}{QFP0}_{vfc}^{h}\left(t\right)-\sum _{v,{c}^{^{\prime}}<c}{QFP1}_{vf{c}^{^{\prime}}}^{h}\left(t\right)}\right]\end{array}\end{array} \end{array}\right.\\ otherwise:0 \end{array}\right.\quad \forall\; v,f,c,h$$

The quantity of vegetable transported by this route accesses a dummy level variable ${V1}_{vfc}^{h}$ (21) which together with the dummy flow variable ${DFP}_{vfc}^{h}$ serves to model the delay in the arrival of the vegetables at the PP during the time equivalent to their transport time. In the dummy variable ${DFP}_{vfc}^{h}$ the Vensim function “DELAY FIXED” is used in which the first element is the quantity of vegetables whose delivery is delayed, and the second element is the amount of time during which the delivery is delayed (22).

21 $${V1}_{vfc}^{h}\left(t\right)=\underset{0}{\overset{t}{\int }}\left({QFP1}_{vfc}^{h}\left(t\right)-{DFP}_{vfc}^{h}\left(t\right)\right)dt;\quad {V1}_{vfc}^{h}\left({t}_{0}\right)=0 \quad\forall \; v,f,c,h$$

22 $${DFP}_{vfc}^{h}\left(t\right)=DELAY\, FIXED\left({QFP1}_{vfc}^{h}\left(t\right), {tfp}_{fc}\left(t\right) \right)\quad \forall \; v,f,c,h$$

Once the vegetables arrive at the PP, they are stored in a pre-packing warehouse, whose inventory ${TIP}_{vc}^{h}$ is calculated as the difference between the vegetables transported from the farms and the quantity of vegetables packed or wasted (23). The value of this variable at the beginning of the simulation is zero.

23 $$\begin{aligned}& {TIP}_{vc}^{h}\left(t\right)=\underset{0}{\overset{t}{\int }}\left[\sum _{f}\left({QFP0}_{vfc}^{h}\left(t\right)+{DFP}_{vfc}^{h}\left(t\right)\right)-{QP}_{vc}^{h}(t)-{WSP}_{vc}^{h}(t)-{WCP}_{vc}^{h}(t) \right]dt; \\ &\quad \qquad{TIP}_{vc}^{h}\left({t}_{0}\right)=0 \forall v,c,h\end{aligned}$$

To determine the quantity of vegetables to be packed ${QP}_{vc}^{h}$, it is checked whether the packing capacity is higher than the quantity of vegetables available for packing (current inventory and new arrivals) (24). If there is sufficient capacity in the PP, all available vegetables are packed. If not, the proportion of each vegetable for which sufficient capacity is available is packed.

24 $${QP}_{vc}^{h}\left(t\right)=\left\{\begin{array}{l}\begin{array}{l}if\quad {pp}_{c}\ge \left(\sum _{v,h}{TIP}_{vc}^{h}\left(t\right)+\sum _{v,f,h}\left({QFP0}_{vfc}^{h}\left(t\right)+{DFP}_{vfc}^{h}\left(t\right)\right)\right) : \\ \left[{TIP}_{vc}^{h}(t)+\sum _{f}\left({QFP0}_{vfc}^{h}(t)+{DFP}_{vfc}^{h}(t)\right)\right]\end{array}\\ otherwise: \left[\frac{\left({TIP}_{vc}^{h}(t)+\sum _{f}\left({QFP0}_{vfc}^{h}(t)+{DFP}_{vfc}^{h}(t)\right)\right)\cdot {pp}_{c}}{\left(\sum _{v,h}{TIP}_{vc}^{h}(t)+\sum _{v,f,h}\left({QFP0}_{vfc}^{h}(t)+{DFP}_{vfc}^{h}(t)\right)\right)}\right]\end{array}\right.\quad \forall \; v,c,h$$

The level variable ${TQP}_{vch}$ consolidates all the vegetables packed in the packing plants for dispatch to warehouses or DCs in the same period of their packing (25). Therefore, this variable must have a value equal to zero in all periods.

25 $$\begin{aligned}& {TQP}_{vc}^{h}\left(t\right)=\underset{0}{\overset{t}{\int }}\left({QP}_{vc}^{h}\left(t\right)-\sum _{w}\left({QPW0}_{vcw}^{h}+{QPW1}_{vcw}^{h}\right)-\sum _{d}\left({QPD0}_{vcd}^{h}+{QPD1}_{vcd}^{h}\right)\right) dt;\\ & {TQP}_{vc}^{h}\left({t}_{0}\right)=0\quad \forall \; v,c,h\end{aligned}$$

Once packed, priority is given to sending the vegetables to the DCs until their handling and storage capacity is full, after which the remaining vegetables are sent to the warehouses. To define the quantity of vegetables ${QPD0}_{vcd}^{h}$ transported to the nearest DC (${tpd}_{cd}=0$), it is checked whether the DC has sufficient capacity to handle the packed vegetables (26). If it is, all the vegetables are transported to the DC and if it is not, only the proportional part of each vegetable is transported. The same logic is applied to the quantity of vegetables transported to DCs with a transport time greater than zero ${QPD1}_{vcd}^{h}$ in case there are still vegetables to be transported (27).

The packed vegetables remaining to be transported are transported first to the closest warehouses (${QPW0}_{vcw}^{h}$) (28) and secondly to warehouses with a transport time greater than zero (${QPW1}_{vcw}^{h}$) (29). The logic used in defining these variables is analogous to the variables ${QPD0}_{vcd}^{h}$ and ${QPD1}_{vcd}^{h}$, respectively.

26 $${QPD0}_{vcd}^{h}\left(t\right)=\left\{\begin{array}{l}if\quad {tpd}_{cd}(t)=0\left\{\begin{array}{l}\begin{array}{l}if\quad \left({hd}_{d}-\sum _{v,{c}^{^{\prime}}<c,h}{QPD0}_{v{c}^{^{\prime}}d}^{h}\left(t\right)\right)\ge \left(\sum_{v,h}{QP}_{vc}^{h}\left(t\right)-\sum _{v,{d}^{^{\prime}}<d,h}{QPD0}_{vc{d}^{^{\prime}}}^{h}\left(t\right)\right) : \\ \left[{QP}_{vc}^{h}(t)-\sum _{{d}^{^{\prime}}<d}{QPD0}_{vc{d}^{^{\prime}}}^{h}(t)\right]\end{array} \\ \begin{array}{l}otherwise: \\ \left[\frac{\left({QP}_{vc}^{h}(t)-\sum _{{d}^{^{\prime}}<d}{QPD0}_{vc{d}^{^{\prime}}}^{h}(t)\right)\cdot \left({hd}_{d}-\sum _{v,{c}^{^{\prime}}<c,h}{QPD0}_{v{c}^{^{\prime}}d}^{h}(t)\right)}{\sum_{v,h}{QP}_{vc}^{h}(t)-\sum _{v,{d}^{^{\prime}}<d,h}{QPD0}_{vc{d}^{^{\prime}}}^{h}(t)}\right]\end{array} \end{array}\right.\\ otherwise: \left[0\right] \end{array}\right.\quad \forall\; v,c,d,h$$

27 $${QPD1}_{vcd}^{h}\left(t\right)=\left\{\begin{array}{l}if\quad {tpd}_{cd}\left(t\right) > 0\left\{\begin{array}{l}\begin{array}{l}if\quad \left({hd}_{d}-\sum _{v,c,h}{QPD0}_{vcd}^{h}\left(t\right)-\sum _{v,{c}^{^{\prime}} < c,h}{QPD1}_{v{c}^{^{\prime}}d}^{h}\left(t\right)\right)\\ \ge \left(\sum_{v,h}{QP}_{vc}^{h}(t)-\sum _{v,d,h}{QPD0}_{vcd}^{h}\left(t\right)-\sum _{v,{d}^{^{\prime}} < d,h}{QPD1}_{vc{d}^{^{\prime}}}^{h}\left(t\right)\right) :\\ \left[{QP}_{vc}^{h}(t)-\sum _{d}{QPD0}_{vcd}^{h}\left(t\right)-\sum _{{d}^{^{\prime}} < d}{QPD1}_{vc{d}^{^{\prime}}}^{h}(t)\right]\end{array}\\ \begin{array}{l}otherwise: \\ \left[\frac{\left({QP}_{vc}^{h}(t)-\sum _{d}{QPD0}_{vcd}^{h}\left(t\right)-\sum _{{d}^{^{\prime}} < d}{QPD1}_{vc{d}^{^{\prime}}}^{h}(t)\right)\cdot \left({hd}_{d}-\sum _{v,c,h}{QPD0}_{vcd}^{h}\left(t\right)-\sum _{v,{c}^{^{\prime}} < c,h}{QPD1}_{v{c}^{^{\prime}}d}^{h}\left(t\right)\right)}{\sum_{v,h}{QP}_{vch}^{h}(t)-\sum _{v,d,h}{QPD0}_{vcd}^{h}\left(t\right)-\sum _{v,{d}^{^{\prime}} < d,h}{QPD1}_{vc{d}^{^{\prime}}}^{h}\left(t\right)}\right]\end{array} \end{array}\right.\\ otherwise: \left[0\right] \end{array}\right.\quad \forall\; v,c,d,h$$

28 $${QPW0}_{vcw}^{h}\left(t\right)=\left\{\begin{array}{l}if\quad {tpw}_{cw}(t)=0\left\{\begin{array}{l}\begin{array}{l}if\quad \left({hw}_{w}-\sum _{v,{c}^{^{\prime}}< c,h}{QPW0}_{v{c}^{^{\prime}}w}^{h}\left(t\right)\right)\\ \ge \left(\sum_{v,h}{QP}_{vc}^{h}\left(t\right)-\sum _{v,d,h}{QPD0}_{vcd}^{h}\left(t\right)-\sum _{v,d,h}{QPD1}_{vcd}^{h}\left(t\right)\right. \\ \left.-\sum _{v,{w}^{^{\prime}}< w,h}{QPW0}_{vc{w}^{^{\prime}}}^{h}(t)\right) : \\ \left[{QP}_{vc}^{h}(t)-\sum _{d}{QPD0}_{vcd}^{h}\left(t\right)-\sum _{d}{QPD1}_{vcd}^{h}\left(t\right)-\sum _{{w}^{^{\prime}} < w}{QPW0}_{vc{w}^{^{\prime}}}^{h}(t)\right]\end{array}\\ \begin{array}{l}otherwise: \\ \left[\frac{\left({QP}_{vc}^{h}(t)-\sum _{d}{QPD0}_{vcd}^{h}\left(t\right)-\sum _{d}{QPD1}_{vcd}^{h}\left(t\right)-\sum _{{w}^{^{\prime}}< w}{QPW0}_{vc{w}^{^{\prime}}}^{h}(t)\right)\cdot \left({hw}_{w}-\sum _{v,{c}^{^{\prime}} < c,h}{QPW0}_{v{c}^{^{\prime}}wh}\left(t\right)\right)}{\sum_{v,h}{QP}_{vch}\left(t\right)-\sum _{v,d,h}{QPD0}_{vcdh}\left(t\right)-\sum _{v,d,h}{QPD1}_{vcdh}\left(t\right)-\sum _{v,{w}^{^{\prime}} < w,h}{QPW0}_{vc{w}^{^{\prime}}h}(t)}\right]\end{array} \end{array}\right.\\ otherwise: \left[0\right] \end{array}\right.\quad \forall\; v,c,w,h$$

29 $${QPW1}_{vcw}^{h}\left(t\right)=\left\{\begin{array}{l}if\quad {tpw}_{cw}\left(t\right)>0\left\{\begin{array}{l}\begin{array}{l}if\quad \left({hw}_{w}-\sum _{v,c,h}{QPW0}_{vcw}^{h}\left(t\right)-\sum _{v,{c}^{^{\prime}}< c,h}{QPW1}_{v{c}^{^{\prime}}w}^{h}\left(t\right)\right)\\\ge \left(\sum_{v,h}{QP}_{vc}^{h}\left(t\right)-\sum _{v,d,h}{QPD0}_{vcd}^{h}\left(t\right)-\sum _{v,d,h}{QPD1}_{vcd}^{h}\left(t\right)-\sum _{v,w,h}{QPW0}_{vcw}^{h}\left(t\right)-\sum _{v,{w}^{^{\prime}}< w,h}{QPW1}_{vc{w}^{^{\prime}}}^{h}\right) : \\ \left[{QP}_{vc}^{h}(t)-\sum _{d}{QPD0}_{vcd}^{h}\left(t\right)-\sum _{d}{QPD1}_{vcd}^{h}\left(t\right)-\sum _{w}{QPW0}_{vcw}^{h}(t)-\sum _{{w}^{^{\prime}}< w}{QPW1}_{vc{w}^{^{\prime}}}^{h}(t)\right]\end{array}\\ \begin{array}{l}otherwise: \\ \left[\frac{\left({QP}_{vc}^{h}(t)-\sum _{d}{QPD0}_{vcd}^{h}\left(t\right)-\sum _{d}{QPD1}_{vcd}^{h}\left(t\right)-\sum _{w}{QPW0}_{vcw}^{h}(t)-\sum _{{w}^{^{\prime}}< w}{QPW1}_{vc{w}^{^{\prime}}}^{h}(t)\right)\cdot \left({hw}_{w}-\sum _{v,c,h}{QPW0}_{vcw}^{h}\left(t\right)-\sum _{v,{c}^{^{\prime}}< c,h}{QPW1}_{v{c}^{^{\prime}}w}^{h}\left(t\right)\right)}{\sum_{v,h}{QP}_{vc}^{h}\left(t\right)-\sum _{v,d,h}{QPD0}_{vcd}^{h}\left(t\right)-\sum _{v,d,h}{QPD1}_{vcd}^{h}\left(t\right)-\sum _{v,w,h}{QPW0}_{vcw}^{h}\left(t\right)-\sum _{v,{w}^{^{\prime}}< w,h}{QPW1}_{vc{w}^{^{\prime}}}^{h}}\right]\end{array} \end{array}\right.\\ otherwise: \left[0\right] \end{array}\right.\quad \forall\; v,c,d,h$$

To ensure that the vegetables shipped between the nodes of the AFSC remain in transit during the transport time, the dummy variables ${V2}_{vcd}^{h}$ (30) and ${DPD}_{vcd}^{h}$ (31) are created for the vegetables transported between the PPs and the DCs, and the dummy variables ${V3}_{vcw}^{h}$ (32) and ${DPW}_{vcw}^{h}$ (33) for the vegetables transported between PPs and warehouses. Their formulation is analogous to that of the variables ${V1}_{vfc}^{h}$ and ${DFP}_{vfc}^{h}$.

30 $${V2}_{vcd}^{h}\left(t\right)=\underset{0}{\overset{t}{\int }}\left({QPD1}_{vcd}^{h}\left(t\right)-{DPD}_{vcd}^{h}\left(t\right)\right)dt;\quad {V2}_{vcd}^{h}\left({t}_{0}\right)=0\quad \forall \; v,c,d,h$$

31 $${DPD}_{vcd}^{h}\left(t\right)=DELAY FIXED\left({QPD1}_{vcd}^{h}\left(t\right), {tpd}_{cd}\left(t\right) \right)\quad \forall \; v,c,d,h$$

32 $${V3}_{vcw}^{h}\left(t\right)=\underset{0}{\overset{t}{\int }}\left({QPW1}_{vcw}^{h}\left(t\right)-{DPW}_{vcw}^{h}\left(t\right)\right)dt;\quad {V3}_{vcw}^{h}\left({t}_{0}\right)=0\quad \forall \; v,c,w,h$$

33 $${DPW}_{vcw}^{h}\left(t\right)=DELAY FIXED\left({QPW1}_{vcw}^{h}\left(t\right), {tpw}_{cw}\left(t\right) \right)\quad \forall \; v,c,w,h$$

When vegetables arrive at the warehouses, they can be stored or transported to the DCs. The inventory at the warehouses ${TIW}_{vw}^{h}$ is calculated as the difference between the vegetables transported from the PPs and those transported to the DCs or wasted (34). The value of this variable at the start of the simulation is zero.

$$\begin{aligned} {TIW}_{vw}^{h}\left(t\right)=\,&\underset{0}{\overset{t}{\int }}\left[\sum _{c}\left({QPW0}_{vcw}^{h}\left(t\right)+{DPW}_{vcw}^{h}\left(t\right)\right)-\sum _{d}\left({QWD0}_{vwd}^{h}\left(t\right)+{QWD1}_{vwd}^{h}\left(t\right)\right)\right. \\& \left.-{WSW}_{vw}^{h}\left(t\right)-{WCW}_{vw}^{h}\left(t\right) \vphantom{\left[\sum _{c}\left({QPW0}_{vcw}^{h}\left(t\right)+{DPW}_{vcw}^{h}\left(t\right)\right)-\sum _{d}\left({QWD0}_{vwd}^{h}\left(t\right)+{QWD1}_{vwd}^{h}\left(t\right)\right)\right.}\right]dt;\end{aligned}$$

34 $${TIW}_{vw}^{h}\left({t}_{0}\right)=0\quad \forall \; v,w,h$$

In turn, the warehouses transport the vegetables to the nearest DC with available vegetable handling capacity. To transport vegetables to a near DC ${QWD0}_{vwd}^{h}$ it is first checked if the transport time is zero (35). If it is, it is checked whether the available handling capacity in the DC is greater than the quantity stored in the warehouse. If yes, all the stored vegetables would be transported, otherwise only the proportional part of each vegetable that can be handled would be transported.

Analogously, if there are still vegetables in the warehouses and to determine the quantity of vegetables to be transported to DCs ${QWD1}_{vwd}^{h}$, it will be checked whether DCs with a transport time greater than zero have sufficient handling capacity to receive more vegetables. If there is, all the vegetables are transported; if there is not enough, only the proportional quantity of each vegetable that can be handled is transported (36).

35 $${QWD0}_{vwd}^{h}\left(t\right)=\left\{\begin{array}{l}if\quad {tiwd}_{cd}\left(t\right)=0\\ \left\{\begin{array}{l}\begin{array}{l}if\quad \left({hd}_{d}-\sum _{v,c,h}\left({QPD0}_{vcd}^{h}\left(t\right)+{DPD}_{vcd}^{h}\left(t\right)\right)-\sum _{v,{w}^{^{\prime}} < w,h}{QWD0}_{v{w}^{^{\prime}}d}^{h}\left(t\right)\right)\\ \ge \left(\sum_{v,h}{TIW}_{vw}^{h}\left(t\right)+\sum _{v,c,h}\left({QPW0}_{vcw}^{h}\left(t\right)+{DPW}_{vcw}^{h}\left(t\right)\right)-\sum _{v,{d}^{^{\prime}}< d,h}{QWD0}_{vw{d}^{^{\prime}}}^{h}\left(t\right)\right) :\\ \left[{TIW}_{vw}^{h}\left(t\right)+\sum _{c}\left({QPW0}_{vcw}^{h}\left(t\right)+{DPW}_{vcw}^{h}(t)\right)-\sum _{{d}^{^{\prime}} < d}{QWD0}_{vw{d}^{^{\prime}}}^{h}\left(t\right)\right]\end{array}\\ \begin{array}{l}otherwise: \\ \left[\frac{\left({TIW}_{vw}^{h}\left(t\right)+\sum _{c}\left({QPW0}_{vcw}^{h}\left(t\right)+{DPW}_{vcw}^{h}(t)\right)-\sum _{{d}^{^{\prime}} < d}{QWD0}_{vw{d}^{^{\prime}}}^{h}\left(t\right)\right)\cdot \left({hd}_{d}-\sum _{v,c,h}{QPD0}_{vcd}^{h}\left(t\right)-\sum _{v,c,h}{DPD}_{vcd}^{h}\left(t\right)-\sum _{v,{w}^{^{\prime}}< w,h}{QWD0}_{v{w}^{^{\prime}}d}^{h}(t)\right)}{\sum_{v,h}{TIW}_{vw}^{h}\left(t\right)+\sum _{v,c,h}{QPW0}_{vcw}^{h}+\sum _{v,c,h}{DPW}_{vcw}^{h}-\sum _{v,{d}^{^{\prime}}< d,h}{QWD0}_{vw{d}^{^{\prime}}}^{h}\left(t\right)}\right]\end{array} \end{array}\right.\\ otherwise:0 \end{array}\right.\quad \forall\; v,w,d,h$$

36 $${QWD1}_{vwd}^{h}\left(t\right)=\left\{\begin{array}{l}if\quad {tiwd}_{cd}\left(t\right)>0\left\{\begin{array}{l}\begin{array}{l}\begin{array}{l}if \left({hd}_{d}-\sum _{v,c,h}\left({QPD0}_{vcd}^{h}\left(t\right)+{DPD}_{vcd}^{h}\left(t\right)\right)-\sum _{v,w,h}{QWD0}_{vwd}^{h}\left(t\right)-\sum _{v,{w}^{^{\prime}}<w,h}{QWD1}_{v{w}^{^{\prime}}d}^{h}\left(t\right)\right)\ge \\ \left(\sum_{v,h}{TIW}_{vw}^{h}\left(t\right)+\sum _{v,c,h}\left({QPW0}_{vcw}^{h}\left(t\right)+{DPW}_{vcw}^{h}(t)\right)-\sum _{v,d,h}{QWD0}_{vwd}^{h}(t)-\sum _{v,{d}^{^{\prime}}<d,h}{QWD1}_{vw{d}^{^{\prime}}}^{h}\left(t\right)\right): \end{array} \\ \left[{TIW}_{vw}^{h}\left(t\right)+\sum _{c}\left({QPW0}_{vcw}^{h}\left(t\right)+{DPW}_{vcw}^{h}(t)\right)-\sum _{d}{QWD0}_{vwd}^{h}\left(t\right)-\sum _{{d}^{^{\prime}}<d}{QWD1}_{vw{d}^{^{\prime}}}^{h}\left(t\right)\right]\end{array}\\ \begin{array}{l}otherwise: \\ \begin{array}{l}\left[\left({TIW}_{vw}^{h}\left(t\right)+\sum _{c}\left({QPW0}_{vcw}^{h}\left(t\right)+{DPW}_{vcw}^{h}(t)\right)-\sum _{d}{QWD0}_{vwd}^{h}\left(t\right)-\sum _{{d}^{^{\prime}}<d}{QWD1}_{vw{d}^{^{\prime}}}^{h}\left(t\right)\right)\right.\cdot \\ \left.\frac{\left({hd}_{d}-\sum _{v,c,h}\left({QPD0}_{vcd}^{h}\left(t\right)+{DPD}_{vcd}^{h}\left(t\right)\right)-\sum _{v,w,h}{QWD0}_{vwd}^{h}\left(t\right)-\sum _{v,{w}^{^{\prime}}<w,h}{QWD1}_{v{w}^{^{\prime}}d}^{h}\left(t\right)\right)}{\sum_{v,h}{TIW}_{vw}^{h}\left(t\right)+\sum _{v,c,h}\left({QPW0}_{vcw}^{h}\left(t\right)+{DPW}_{vcw}^{h}(t)\right)-\sum _{v,d,h}{QWD0}_{vwd}^{h}(t)-\sum _{v,{d}^{^{\prime}}<d,h}{QWD1}_{vw{d}^{^{\prime}}}^{h}\left(t\right)}\right]\end{array}\end{array} \end{array}\right.\\ otherwise: 0 \end{array}\right.\quad \forall\; v,w,d,h$$

In addition, it is necessary to create the dummy level variable ${V4}_{vwd}^{h}$ (37) which together with the dummy flow variable ${DWD}_{vwd}^{h}$ (36) model the delay in the arrival of the vegetable at the DC during the transport time from warehouses.

37 $${V4}_{vwd}^{h}\left(t\right)=\underset{0}{\overset{t}{\int }}\left({QWD1}_{vwd}^{h}\left(t\right)-{DWD}_{vwd}^{h}\left(t\right)\right)dt;\quad {V4}_{vwd}^{h}\left({t}_{0}\right)=0\quad \forall\; v,w,d,h$$

38 $${DWD}_{vwd}^{h}\left(t\right)=DELAY FIXED\left({QWD1}_{vwd}^{h}\left(t\right), {tiwd}_{wd}\left(t\right) \right)\quad \forall \; v,w,d,h$$

Once they arrive to the DC, vegetables are stored or consolidated to be transported to markets. This inventory ${TID}_{vd}^{h}$ is calculated as the difference between the vegetable transported from PPs and warehouses and transported to markets or wasted (39). The value of this variable at the beginning of the simulation is zero.

$${TID}_{vd}^{h}\left(t\right)=\underset{0}{\overset{t}{\int }}\left[\sum _{c}\left({QPD0}_{vcd}^{h}\left(t\right)+{DPD}_{vcd}^{h}\left(t\right)\right)+\sum _{w}\left({QWD0}_{vwd}^{h}\left(t\right)+{DWD}_{vwd}^{h}\left(t\right)\right)-{WSD}_{vd}^{h}\left(t\right)-{WCD}_{vd}^{h}\left(t\right)\right.$$

39 $$\left.-\sum _{m}\left({QDM0}_{vdm}^{h}\left(t\right)+{QDM1}_{vdm}^{h}\left(t\right)\right)\right]dt;\quad {TID}_{vd}^{h}\left({t}_{0}\right)=0 \forall v,d,h$$

The DCs transport vegetables to all markets, regardless of whether they are close or far away. Thus, ${QDM0}_{vdm}^{h}$ defines the quantity of vegetables to be transported between the DCs and the markets when the transport time is zero (40), and ${QDM1}_{vdm}^{h}$ does the same for the case where the transport time is greater than zero (41). In the last, it is necessary to define the dummy variables ${V5}_{vdm}^{h}$ and ${DDM}_{vdm}^{h}$ to delay the delivery of the vegetables during the transport time between the DC and the market (42) and (43).

40 $${QDM0}_{vdm}^{h}\left(t\right)=\left\{\begin{array}{l}if\quad {tdm}_{dm}=0: \\ \left[\frac{{TID}_{vd}^{h}\left(t\right)+\sum _{c}\left({QPD0}_{vcd}^{h}\left(t\right)+{DPD}_{vcd}^{h}\left(t\right)\right)+\sum _{w}\left({QWD0}_{vwd}^{h}\left(t\right)+{DWD}_{vwd}^{h}\left(t\right)\right)}{nm}\right]\\ otherwise: \left[0\right] \end{array}\right.\quad\forall\; v,d,m,h$$

41 $${QDM1}_{vdm}^{h}\left(t\right)=\left\{\begin{array}{l}if\quad {tdm}_{dm}>0: \\ \left[\frac{{TID}_{vd}^{h}\left(t\right)+\sum _{c}\left({QPD0}_{vcd}^{h}\left(t\right)+{DPD}_{vcd}^{h}\left(t\right)\right)+\sum _{w}\left({QWD0}_{vwd}^{h}\left(t\right)+{DWD}_{vwd}^{h}\left(t\right)\right)}{nm}\right]\\ otherwise: \left[0\right] \end{array}\right.\quad\forall\; v,d,m,h$$

42 $${V5}_{vdm}^{h}\left(t\right)=\underset{0}{\overset{t}{\int }}\left({QDM1}_{vdm}^{h}\left(t\right)-{DDM}_{vdm}^{h}\left(t\right)\right)dt;\quad {V5}_{vdm}^{h}\left({t}_{0}\right)=0\quad \forall\; v,d,m,h$$

43 $${DDM}_{vdm}^{h}\left(t\right)=DELAY FIXED\left({QDM1}_{vdm}^{h}\left(t\right), {tdm}_{dm}\left(t\right) \right)\quad \forall \; v,d,m,h$$

The inventory of vegetables in the markets ${TIM}_{vm}^{h}$ is the difference between the vegetables received from the DCs, the vegetables sold, and the waste (44). Its value at the beginning of the simulation is zero. To define he quantity of vegetable sold to end consumers $\left({QS}_{vm}^{h}\right)$ it is checked whether the market has enough vegetable to satisfy the demand. If so, the quantity demanded is sold and if not, the quantity available is sold (45).

44 $${TIM}_{vm}^{h}\left(t\right)=\underset{0}{\overset{t}{\int }}\left(\sum _{d}\left({QDM0}_{vdm}^{h}\left(t\right)+{QDM1}_{vdm}^{h}\left(t\right)\right)-{QS}_{vm}^{h}-{WSM}_{vm}^{h}\right) dt;\quad {TIM}_{vm}^{h}\left({t}_{0}\right)=0\quad \forall \; v,m,h$$

45 $${QS}_{vm}^{h}\left(t\right)=\left\{\begin{array}{l}if\quad {D}_{vm}>0: \left\{\begin{array}{l}if \sum _{h}\left({TIM}_{vm}^{h}+\sum _{d}\left({QDM0}_{vdm}^{h}\left(t\right)+{DDM}_{vdm}^{h}\left(t\right)\right)\right)\ge {D}_{vm}: \\ \left[\frac{\left({TIM}_{vm}^{h}+\sum _{d}\left({QDM0}_{vdm}^{h}\left(t\right)+{DDM}_{vdm}^{h}\left(t\right)\right)\right)\cdot \left({D}_{vm}\right)}{\sum _{h}\left({TIM}_{vm}^{h}+\sum _{d}\left({QDM0}_{vdm}^{h}\left(t\right)+{DDM}_{vdm}^{h}\left(t\right)\right)\right)}\right]\\ otherwise: \\ \left[{TIM}_{vm}^{h}+\sum _{d}\left({QDM0}_{vdm}^{h}\left(t\right)+{DDM}_{vdm}^{h}\left(t\right)\right)\right] \end{array}\right.\\ otherwise: \left[0\right] \end{array}\right. \quad\forall \; v,m,h$$

If the vegetables stored in the PPs (46), warehouses (47), DCs (48) or markets (49) consume their shelf-life to a shorter shelf-life than that required by the end consumers, they are wasted.

46 $${WSP}_{vc}^{h}\left(t\right)=\left\{\begin{array}{l}if\quad t>\left({th}_{v}\left(t\right)+sl-msl\right) : \\ \left[{TIP}_{vc}^{h}\left(t\right)+\sum _{f}\left({QFP0}_{vfc}^{h}\left(t\right)+{DFP}_{vfc}^{h}\left(t\right)\right)-{QP}_{vc}^{h}\right]\\ otherwise: 0 \end{array}\right.\quad \forall\; v,c,h$$

47 $${WSW}_{vw}^{h}\left(t\right)=\left\{\begin{array}{l}if\quad t>\left({th}_{v}\left(t\right)+sl-msl\right) : \\ \left[{TIW}_{vw}^{h}\left(t\right)+\sum _{c}\left({QPW0}_{vcw}^{h}\left(t\right)+{DPW}_{vcw}^{h}\left(t\right)\right)-\sum _{d}\left({QWD0}_{vwd}^{h}\left(t\right)+{QWD1}_{vwd}^{h}\left(t\right)\right)\right]\\ otherwise: 0 \end{array}\right.\quad \forall \; v,w,h$$

48 $${WSD}_{vd}^{h}\left(t\right)=\left\{\begin{array}{l}if\quad t>\left({th}_{v}\left(t\right)+sl-msl\right) : \\ \left[{TID}_{vd}^{h}\left(t\right)+\sum _{c}\left({QPD0}_{vcd}^{h}\left(t\right)+{DPD}_{vcd}^{h}\left(t\right)\right)+\sum _{w}\left({QWD0}_{vwd}^{h}\left(t\right)+{DWD}_{vwd}^{h}\left(t\right)\right)\right.\\ \left.-\sum _{d}\left({QDM0}_{vdm}^{h}\left(t\right)+{QDM1}_{vdm}^{h}\left(t\right)\right)\right] \\ otherwise: 0 \end{array}\right.\quad\forall\; v,d,h$$

49 $${WSM}_{vm}^{h}\left(t\right)=\left\{\begin{array}{l}if\quad t>\left({th}_{v}\left(t\right)+sl-msl\right) : \\ \left[{TIM}_{vm}^{h}\left(t\right)+\sum _{d}\left({QDM0}_{vdm}^{h}\left(t\right)+{DDM}_{vdm}^{h}\left(t\right)\right)-{QS}_{cm}^{h}\right]\\ otherwise: 0 \end{array}\right. \quad\forall \;v,m,h$$

Analogously, if once vegetables are wasted due to perishability reasons more vegetables arrive at PPs (50), warehouses (51) or DCs (52) than can be packed, handled, and stored in each period, the excess vegetables are wasted proportionally at the PPs (${WCP}_{vc}^{h}$), warehouses (${WCW}_{vw}^{h}$) or DCs (${WCD}_{vd}^{h}$) respectively.

50 $${WCP}_{vc}^{h}\left(t\right)=\left\{\begin{array}{l}\begin{array}{l}if\quad {sp}_{c}<\sum _{v,h}{TIP}_{vc}^{h}(t)+\sum _{v,f,h}\left({QFP0}_{vfc}^{h}\left(t\right)+{DFP}_{vfc}^{h}\left(t\right)\right)-\sum _{v,h}{QP}_{vc}^{h}\left(t\right)-\sum _{v,h}{WSP}_{vc}^{h}\left(t\right) : \\ \begin{array}{l}\left[\left({TIP}_{vc}^{h}(t)+\sum _{f}\left({QFP0}_{vfc}^{h}\left(t\right)+{DFP}_{vfc}^{h}\left(t\right)\right)-{QP}_{vch}^{h}\left(t\right)-{WSP}_{vc}^{h}\left(t\right)\right)\cdot \right.\\ \left.\frac{\left(\sum _{v,h}{TIP}_{vc}^{h}(t)+\sum _{v,f,h}\left({QFP0}_{vfc}^{h}\left(t\right)+{DFP}_{vfc}^{h}\left(t\right)\right)-\sum _{v,h}{QP}_{vc}^{h}\left(t\right)-\sum _{v,h}{WSP}_{vc}^{h}\left(t\right)-{sp}_{c}\right)}{\sum _{v,h}{TIP}_{vc}^{h}(t)+\sum _{v,f,h}\left({QFP0}_{vfc}^{h}\left(t\right)+{DFP}_{vfc}^{h}\left(t\right)\right)-\sum _{v,h}{QP}_{vc}^{h}\left(t\right)-\sum _{v,h}{WSP}_{vc}^{h}\left(t\right)}\right]\end{array}\end{array}\\ otherwise: 0 \end{array}\right.\quad \forall \; v,c,h$$

51 $$\begin{aligned}& {WCW}_{vw}^{h}\left(t\right)\\ &=\left\{\begin{array}{l}\begin{array}{l}if\quad {sw}_{w}\\<\sum _{v,h}\left({TIW}_{vw}^{h}\left(t\right)+\sum _{c}\left({QPW0}_{vcw}^{h}\left(t\right)+{DPW}_{vcw}^{h}\left(t\right)\right)-\sum _{d}\left({QWD0}_{vwd}^{h}\left(t\right)+{QWD1}_{vwd}^{h}\left(t\right)\right)-{WSW}_{vw}^{h}\left(t\right)\right) : \\ \begin{array}{l}\left[\left({TIW}_{vw}^{h}\left(t\right)+\sum _{c}\left({QPW0}_{vcw}^{h}\left(t\right)+{DPW}_{vcw}^{h}\left(t\right)\right)-\sum _{d}\left({QWD0}_{vwd}^{h}\left(t\right)+{DWD}_{vwd}^{h}\left(t\right)\right)-{WSW}_{vw}^{h}\left(t\right)\right)\right.\cdot \\ \left.\frac{\left(\sum _{v,h}\left({TIW}_{vw}^{h}\left(t\right)+\sum _{c}\left({QPW0}_{vcw}^{h}\left(t\right)+{DPW}_{vcw}^{h}\left(t\right)\right)-\sum _{d}\left({QWD0}_{vwd}^{h}\left(t\right)+{DWD}_{vwd}^{h}\left(t\right)\right)-{WSW}_{vw}^{h}\left(t\right)\right)-{sw}_{w}\right)}{\sum _{v,h}\left({TIW}_{vw}^{h}\left(t\right)+\sum _{c}\left({QPW0}_{vcw}^{h}\left(t\right)+{DPW}_{vcw}^{h}\left(t\right)\right)-\sum _{d}\left({QWD0}_{vwd}^{h}\left(t\right)+{DWD}_{vwd}^{h}\left(t\right)\right)-{WSW}_{vw}^{h}\left(t\right)\right)}\right]\end{array}\end{array}\\ otherwise: 0 \end{array}\right.\quad\forall\; v,w,h\end{aligned}$$

52 $${WCD}_{vd}^{h}\left(t\right)=\left\{\begin{array}{l}\begin{array}{l}if\quad {sd}_{d}<\sum _{v,h}\left({TID}_{vd}^{h}\left(t\right)+\sum _{c}\left({QPD0}_{vcd}^{h}\left(t\right)+{DPD}_{vcd}^{h}\left(t\right)\right)+\sum _{w}\left({QWD0}_{vwd}^{h}\left(t\right)+{DWD}_{vwd}^{h}\left(t\right)\right)\right. \\ \left.-\sum _{d}\left({QDM0}_{vdm}^{h}\left(t\right)+{QDM1}_{vdm}^{h}\left(t\right)\right)-{WSD}_{vd}^{h}\left(t\right)\right) :\\ \begin{array}{l}\left[\left({TID}_{vd}^{h}\left(t\right)+\sum _{c}\left({QPD0}_{vcd}^{h}\left(t\right)+{DPD}_{vcd}^{h}\left(t\right)\right)+\sum _{w}\left({QWD0}_{vwd}^{h}\left(t\right)+{DWD}_{vwd}^{h}\left(t\right)\right)\right. \right. \\ \left. \left.-\sum _{d}\left({QDM0}_{vdm}^{h}\left(t\right)+{QDM1}_{vdm}^{h}\left(t\right)\right)-{WSD}_{vd}^{h}\left(t\right)\right)\right.\cdot \\ \left.\frac{\left(\sum _{v,h}\left({TID}_{vd}^{h}\left(t\right)+\sum _{c}\left({QPD0}_{vcd}^{h}\left(t\right)+{DPD}_{vcd}^{h}\left(t\right)\right)+\sum _{w}\left({QWD0}_{vwd}^{h}\left(t\right)+{DWD}_{vwd}^{h}\left(t\right)\right)-\sum _{d}\left({QDM0}_{vdm}^{h}\left(t\right)+{QDM1}_{vdm}^{h}\left(t\right)\right)-{WSD}_{vd}^{h}\left(t\right)\right)-{sd}_{d}\right)}{\sum _{v,h}\left({TID}_{vd}^{h}\left(t\right)+\sum _{c}\left({QPD0}_{vcd}^{h}\left(t\right)+{DPD}_{vcd}^{h}\left(t\right)\right)+\sum _{w}\left({QWD0}_{vwd}^{h}\left(t\right)+{DWD}_{vwd}^{h}\left(t\right)\right)-\sum _{d}\left({QDM0}_{vdm}^{h}\left(t\right)+{QDM1}_{vdm}^{h}\left(t\right)\right)-{WSD}_{vd}^{h}\left(t\right)\right)}\right]\end{array}\end{array}\\ otherwise: 0 \end{array}\right.\quad\forall\; v,d,h$$

The cumulative quantity of wasted vegetables due to consumption of their shelf-life or due to lack of capacity of the PPs (53), warehouses (54), DCs (55), and markets (56) is collected in level variables ${TWP}_{vch}$, ${TWW}_{vw}^{h}$, ${TWD}_{vd}^{h}$, and ${TWM}_{vm}^{h}$ respectively. The value of these variables at the beginning of the simulation is zero.

53 $${TWP}_{vc}^{h}\left(t\right)=\underset{0}{\overset{t}{\int }}\left({WSP}_{vc}^{h}\left(t\right)+{WCP}_{vc}^{h}\left(t\right)\right) dt;\quad {TWP}_{vc}^{h}\left({t}_{0}\right)=0\quad \forall\; v,c,h$$

54 $${TWW}_{vw}^{h}\left(t\right)=\underset{0}{\overset{t}{\int }}\left({WSW}_{vw}^{h}\left(t\right)+{WCW}_{vw}^{h}\left(t\right)\right) dt;\quad {TWW}_{vw}^{h}\left({t}_{0}\right)=0 \quad\forall\; v,w,h$$

55 $${TWD}_{vd}^{h}\left(t\right)=\underset{0}{\overset{t}{\int }}\left({WSD}_{vd}^{h}\left(t\right)+{WCD}_{vd}^{h}\left(t\right)\right) dt;\quad {TWD}_{vd}^{h}\left({t}_{0}\right)=0 \quad\forall\; v,d,h$$

56 $${TWM}_{vm}^{h}\left(t\right)=\underset{0}{\overset{t}{\int }}\left({WSM}_{vm}^{h}\left(t\right)\right) dt;\quad {TWM}_{vm}^{h}\left({t}_{0}\right)=0 \quad\forall \; v,m,h$$

In terms of transport, the number of trucks used to transport vegetables per period between two SC nodes depends on the quantity to be transported and the capacity of the trucks. The variables ${NFP}_{fc}$, ${NPD}_{cd}$, ${NPW}_{cw}$, ${NWD}_{wd}$, and ${NDM}_{dm}$ define the number of trucks used between farms and PPs (57), PPs and DCs (58), PPs and warehouses (59), warehouses and DCs (60), and DCs and markets (61) respectively.

57 $${NFP}_{fc}\left(t\right)=INT\left(\frac{\sum _{v,h}\left({QFP0}_{vfc}^{h}\left(t\right)+{QFP1}_{vfc}^{h}\left(t\right)\right)}{mtl}\right) \quad\forall\; f,c$$

58 $${NPD}_{cd}\left(t\right)=INT\left(\frac{\sum _{v,h}\left({QPD0}_{vpd}^{h}(t)+{QPD1}_{vpd}^{h}(t)\right)}{mtl}\right) \quad\forall\; c,d$$

59 $${NPW}_{cw}\left(t\right)=INT\left(\frac{\sum _{v,h}\left({QPW0}_{vcw}^{h}(t)+{QPW1}_{vcw}^{h}(t)\right)}{mtl}\right)\quad \forall\; c,w$$

60 $${NWD}_{wd}\left(t\right)=INT\left(\frac{\sum _{v,h}\left({QWD0}_{vwd}^{h}(t)+{QWD1}_{vwd}^{h}(t)\right)}{mtl}\right) \quad\forall\; w,d$$

61 $${NDM}_{dm}\left(t\right)=INT\left(\frac{\sum _{v,h}\left({QDM0}_{vdm}^{h}(t)+{QDM1}_{vdm}^{h}(t)\right)}{mtl}\right) \quad\forall\; d,m$$

The total number of trucks used between farms and PPs (62), PPs and DCs (63), PPs and warehouses (64), warehouses and DCs (65), and DCs and markets (66 are defined in level variables, whose values at the start of the simulation is zero.

62 $${TTFP}_{fc}\left(t\right)=\underset{0}{\overset{t}{\int }}\left({NFP}_{fc}\left(t\right)\right) dt;\quad {TTFP}_{fc}\left({t}_{0}\right)=0 \quad\forall \; f,c$$

63 $${TTPD}_{cd}\left(t\right)=\underset{0}{\overset{t}{\int }}\left({NPD}_{cd}\left(t\right)\right) dt;\quad {TTPD}_{cd}\left({t}_{0}\right)=0 \quad\forall \; c,d$$

64 $${TTPW}_{cw}\left(t\right)=\underset{0}{\overset{t}{\int }}\left({NPW}_{cw}\left(t\right)\right) dt;\quad {TTPW}_{cw}\left({t}_{0}\right)=0 \quad\forall\; c,w$$

65 $${TTWD}_{wd}\left(t\right)=\underset{0}{\overset{t}{\int }}\left({NWD}_{wd}\left(t\right)\right) dt;\quad {TTWD}_{wd}\left({t}_{0}\right)=0 \quad\forall\; w,d$$

66 $${TTDM}_{dm}\left(t\right)=\underset{0}{\overset{t}{\int }}\left({NDM}_{dm}\left(t\right)\right) dt;\quad {TTDM}_{dm}\left({t}_{0}\right)=0 \quad\forall\; d,m$$

The demand flow variable ${D}_{vm}$ acquires its value from the auxiliary variable ${dem}_{vm}$ which is an input (67). Satisfied demand ${SD}_{vm}$ is the quantity of vegetable sold on the markets (68). Unsatisfied demand ${UD}_{vm}$ occurs when demand is greater than the quantity sold and is the difference between demand and satisfied demand (69).

67 $${D}_{vm}\left(t\right)={dem}_{vm}\left(t\right) \quad\forall \; v,m$$

68 $${SD}_{vm}\left(t\right)=\sum _{h}{QS}_{vm}^{h}\left(t\right) \quad\forall\; v,m$$

69 $${UD}_{vm}\left(t\right)=\left\{\begin{array}{l}if\quad {D}_{vm}\left(t\right)>{SD}_{vm}\left(t\right): \left[{D}_{vm}\left(t\right)-{SD}_{vm}\left(t\right)\right]\\ otherwise: \left[0\right] \end{array}\right. \quad\forall\; v,m$$

The level variable ${TD}_{vm}\left(t\right)$ represents the balance between the demand of the period and the satisfied and unsatisfied demand of the same period (70). As all the demand must be either satisfied or unsatisfied and it is not possible to backlog demand, this variable must have a value equal to zero in all periods of the simulation.

70 $${TD}_{vm}\left(t\right)=\underset{0}{\overset{t}{\int }}\left({D}_{vm}\left(t\right)-\sum _{h}\left({SD}_{vm}^{h}\left(t\right)-{UD}_{vm}^{h}\left(t\right)\right)\right) dt;\quad {TD}_{vm}\left({t}_{0}\right)=0 \quad\forall\; v,m$$

The total satisfied demand ${TSD}_{vm}$ and total unsatisfied demand ${TUD}_{vm}$ over the horizon is the accumulation of satisfied (71) and unsatisfied (72) demand in each period respectively. The value of these variables at the beginning of the simulation is zero.

71 $${TSD}_{vm}\left(t\right)=\underset{0}{\overset{t}{\int }}\left({SD}_{vm}\left(t\right)\right)dt;\quad {TSD}_{vm}\left({t}_{0}\right)=0 \quad\forall\; v,m$$

72 $${TUD}_{vm}\left(t\right)=\underset{0}{\overset{t}{\int }}\left({UD}_{vm}\left(t\right)\right)dt;\quad {TUD}_{vm}\left({t}_{0}\right)=0 \quad\forall\; v,m$$

As for the economic variables, AFSC profits are calculated as the difference between sales and costs related to planting, cultivating, harvesting, packing, transport, storage and handling of vegetables, hiring of permanent workers, wages of permanent and temporary workers, and economic penalties for wasting vegetables and not serving demand (73). The profit at the beginning of the simulation is zero.

73 $$\begin{aligned} Profit \left(t\right)& =\underset{0}{\overset{t}{\int }}\left(Inc-CostPCH-CostHPL-CostPL-CostTL-CostP-CostFP\right. \\ & \left. \quad-CostPW-CostPD-CostTWD-CostDM-CostIP-CostIW-CostID-CostHW\right. \\ & \left. \quad-CostHD-CostWP-CostWW-CostWD-CostWM-CostUD\right)dt; Profit \left({t}_{0}\right)=0\end{aligned}$$

Sales ($Inc$) are calculated as the quantity of vegetables sold in each market multiplied by the price of the vegetables in those markets (74).

74 $$Inc \left(t\right)=\sum _{v,m,h}{QS}_{vm}^{h}\left(t\right)\cdot {p}_{vm}\left(t\right)$$

Planting, cultivation and harvesting costs ($CostPCH$) are calculated as the area planted with each vegetable on each farmer multiplied by the unit cost of planting, cultivation, and harvesting (75).

75 $$CostPCH \left(t\right)=\sum _{v,f,p}{AP}_{vf}^{p}\left(t\right)\cdot cpch$$

The cost of hiring permanent workers ($CostHPL$) depends on the number of permanent workers and the cost of hiring a permanent worker (76). The wage cost of permanent ($CostPL$) (77) and temporary workers ($CostTL$) (78) depends on the number of workers of each type on the payroll and their weekly wage.

76 $$CostHPL \left(t\right)=\sum _{f}{HPL}_{f}\left(t\right)\cdot chpl$$

77 $$CostPL \left(t\right)=\sum _{f}{TPL}_{f}\left(t\right)\cdot cpl$$

78 $$CostTL \left(t\right)=\sum _{f}{TTL}_{f}\left(t\right)\cdot ctl$$

The cost of packing vegetables ($CostP$) is the quantity of vegetables packed in the different PPs multiplied by the cost of packing one kilogram of vegetable (79).

79 $$CostP \left(t\right)=\sum _{v,c,h}{QP}_{vc}^{h}(t)\cdot {cp}_{v}$$

The transport cost between farms and PP ($CostFP$) (80), PPs and DCs ($CostPD$) (81), PPs and warehouses ($CostPW$) (82), warehouses and DCs ($CostTWD$) (83), and DCs and markets ($CostDM$) (84) depends on the number of trucks used to transport vegetables between two SC nodes and the cost of a truck to make that journey.

80 $$CostFP \left(t\right)=\sum _{f,c}{NFP}_{fc}(t)\cdot cf{p}_{fc}$$

81 $$CostPD \left(t\right)=\sum _{c,d}{NPD}_{cd}(t)\cdot cp{d}_{cd}$$

82 $$CostPW \left(t\right)=\sum _{c,w}{NPW}_{cw}(t)\cdot cp{w}_{cw}$$

83 $$CostTWD \left(t\right)=\sum _{w,d}{NWD}_{wd}(t)\cdot ctw{d}_{wd}$$

84 $$CostDM \left(t\right)=\sum _{d,m}{NDM}_{dm}(t)\cdot cd{m}_{dm}$$

The cost of storing vegetables is calculated as the quantity of vegetables stored in PPs ($CostIP$) (85), warehouses ($CostIW$) (86) and DCs ($CostID$) (87) multiplied by the unit cost of storing vegetables in these nodes.

85 $$CostIP \left(t\right)=\sum _{v,c,h}{TIP}_{vc}^{h}(t)\cdot {cip}_{v}$$

86 $$CostIW \left(t\right)=\sum _{v,w,h}{TIW}_{vw}^{h}(t)\cdot {ciw}_{v}$$

87 $$CostID \left(t\right)=\sum _{v,d,h}{TID}_{vd}^{h}(t)\cdot {cid}_{v}$$

The cost of handling vegetables in warehouses ($CostHW$) (88) and DCs ($CostHD$) (89) depends on the quantity of vegetables arriving at these nodes and the unit cost of handling vegetables at these nodes.

88 $$CostHW \left(t\right)=\sum _{v,c,w,h}\left({QPW0}_{vcw}^{h}(t)+{DPW}_{vcw}^{h}(t)\right)\cdot {chw}_{v}$$

89 $$CostHD \left(t\right)=\sum _{v,w,h}\left({QPD0}_{vcd}^{h}\left(t\right)+{DPD}_{vcd}^{h}\left(t\right)+{QWD0}_{vwd}^{h}(t)+{DWD}_{vwd}^{h}(t)\right)\cdot {chd}_{v}$$

The cost of wasting vegetables is calculated as the quantity of vegetables wasted in PPs ($CostWP$) (90), warehouses ($CostWW$) (91), DCs ($CostWD$) (92), and markets ($CostWM$) (93) multiplied by the economic penalty for wasting vegetable.

90 $$CostWP \left(t\right)=\sum _{v,c,h}\left({WSP}_{vc}^{h}(t)+{WCP}_{vc}^{h}(t)\right)\cdot {cwp}_{v}$$

91 $$CostWW \left(t\right)=\sum _{v,w,h}\left({WSW}_{vw}^{h}(t)+{WCW}_{vw}^{h}(t)\right)\cdot {cww}_{v}$$

92 $$CostWD \left(t\right)=\sum _{v,d,h}\left({WSD}_{vd}^{h}(t)+{WCD}_{vd}^{h}(t)\right)\cdot {cwd}_{v}$$

93 $$CostWM \left(t\right)=\sum _{v,m,h}{WSM}_{vm}^{h}(t)\cdot {cwm}_{v}$$

Finally, the cost of not satisfying vegetable demand ($CostUD$) depends on the quantity of unsatisfied demand and the economic penalty of not serving one kilogram of vegetable demand in the markets (94).

94 $$CostUD \left(t\right)=\sum _{v,m}{UD}_{vm}(t)\cdot {cud}_{v}$$

### Validation of the system dynamics model

The known behavior reproduction test and the extreme conditions test proposed by Sterman (2000) are performed to validate the proposed system dynamics model.

In the known behavior reproduction test, the results obtained by the system dynamics model with the Vensim® software are compared with the results obtained by the equivalent mixed integer linear programming model presented in Esteso et al. (2021) with the MPL® software. To force the system dynamics model to make the optimal decisions for the AFSC, the mathematical programming model is run first, and then the optimal values of the quantity of vegetables to be transported between the nodes of the SC are assigned to the system dynamics model as input data.

Table 4 shows the main results obtained by both models, which are very similar except in the case of planting, cultivating, and harvesting costs. This is because the Vensim® software considers a smaller number of decimals, being necessary to round up the values of the area planted with each vegetable on each farm in each planting period.

**Table 4:** Results of the known behavior reproduction test

	Mathematical programming	System dynamics	Variation (%)
Planting, cultivation, and harvesting costs (€)	1,913,987	1,919,081	0.27
Labor costs (€)	781,754	781,754	0.00
Packing costs (€)	4,020,242	4,020,242	0.00
Storage costs (€)	74,668	74,668	0.00
Handling costs (€)	2,854,307	2,854,305	0.00
Transport costs (€)	4,149,606	4,149,619	0.00
Waste costs (€)	16,672	16,672	0.00
Unsatisfied demand costs (€)	1,315,757	1,315,756	0.00
Sales (€)	65,995,162	65,995,163	0.00
Profit (€)	36,642,967	36,637,864	−0.01
Quantity wasted (kg)	606,941	606,941	0.00
Quantity harvested (kg)	120,872,199	120,877,035	0.00
Quantity sold (kg)	120,265,258	120,265,259	0.00
Unsatisfied demand (kg)	4,610,927	4,610,928	0.00

It is remarkable that the mathematical programming model proposed in Esteso et al. (2021) takes more than five hours to obtain the optimal solution for this AFSC, while the simulation model proposed in this paper give solutions in seconds, thus allowing the rapid assessment of the robustness of the AFSC to several scenarios in very short time.

**Fig. 4:**
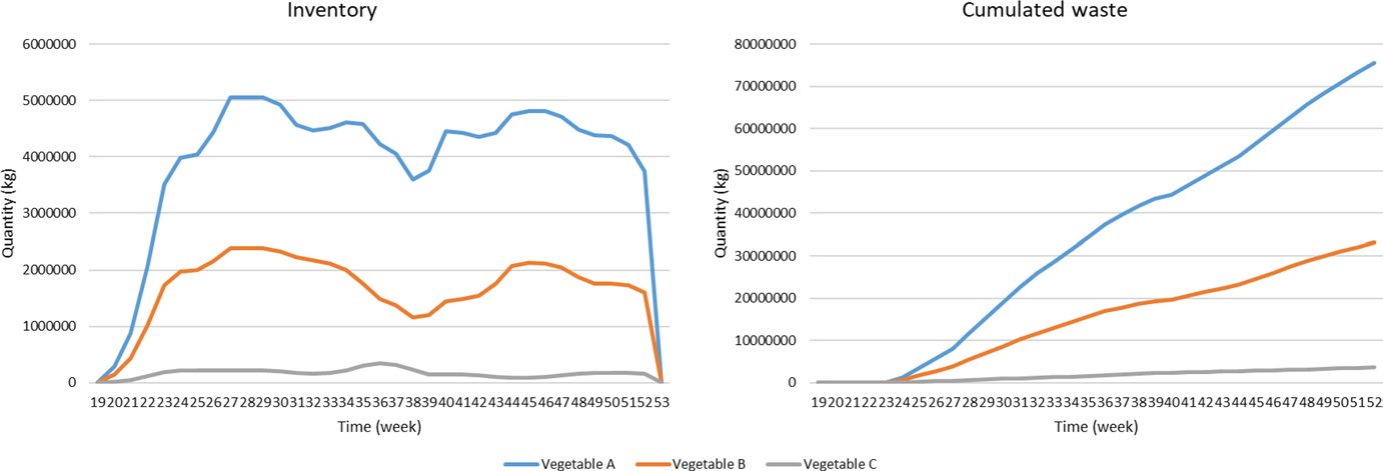
Inventory and wastage accumulated in the no-demand scenario

In the extreme conditions test, two scenarios are considered. In the first scenario, it is considered that there is no demand for any of the vegetables, so it is expected that the harvested vegetables will be packed and moved along the AFSC or kept stored until their shelf-life is used up, at which point they will be wasted. After running this scenario, Fig. 4 shows how the quantity of vegetables stored in the AFSC fluctuates due to the harvesting of vegetables (inventory increase) and the consumption of the shelf-life of the stored vegetables (inventory decrease) and how vegetable wastage increases over the simulation horizon. Note that this figure shows these values from period 19 which is the first week in which vegetables can be harvested.

**Fig. 5:**
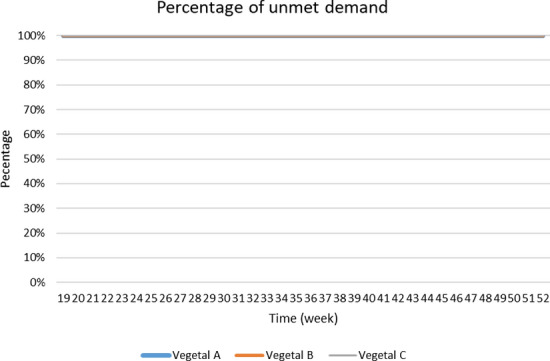
Percentage of unsatisfied demand in the scenario without planting vegetables

The second scenario assumes that no vegetables are planted during the simulation horizon so it is expected that there will be no flow of vegetables through the AFSC since no vegetable harvest will occur. After running this scenario, Fig. 5 shows the percentage of unsatisfied demand in each period is equal to 100%, verifying that no demand can be served due to the shortage of vegetables.

After performing the known behavior reproduction test and the extreme conditions test it is concluded that the proposed system dynamics model is validated.

## Methodology for improving the AFSC robustness to disruptions

**Fig. 6:**
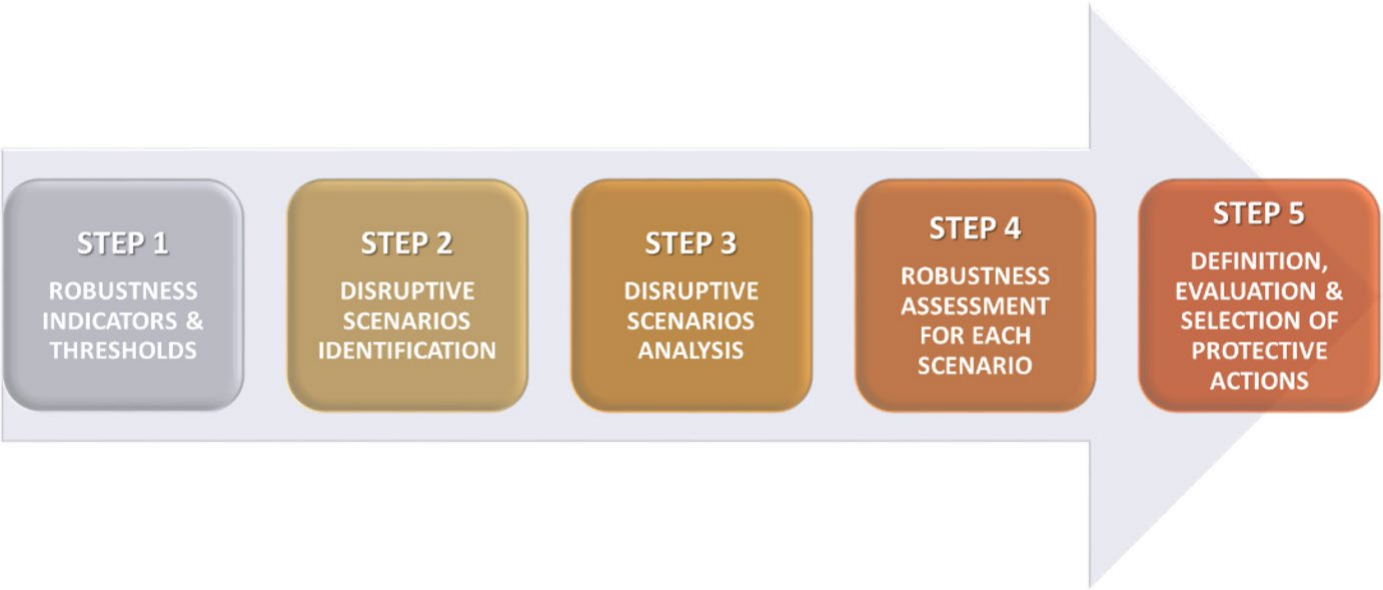
Methodology for improving the AFSC robustness to disruptions

This section, describes the methodology proposed to guide in the use of the SD model in order to assess and boost the level of robustness of an AFSC and its planting planning to disruptions. The proposed methodology consists of the following five stages (Fig. 6):

1. *Definition of robustness indicators and their thresholds* The first step requires the establishment of the most relevant indicators jointly with their permissible limits of variation to consider the AFSC to be robust. It should be noted that the AFSC robustness is a subjective measure that depends on the risk aversion of the SC members. Therefore, the members of the SC should set what they consider to be tolerable limits on SC performance indicators such as SC profits, vegetable wastage generated, satisfied or unsatisfied demand, among others. The greater the risk aversion, the tighter the variation limits.
2. *Disruptive scenarios identification* Possible risks or disruptions should be foreseen with the aim of devising future scenarios. It is assumed that a specific scenario is defined by the disruption types considered (e.g.: demand and supply) and for each type, the level of the disruption (e.g.: 1.25 normal demand and 0.5*normal supply). One scenario can include none, one or multiple disruption types. The scenario not including any disruption type is named baseline scenario and represents the SC regular or as usual operating mode. It is taken as a benchmark when evaluating the consequences of disruptive scenarios.
3. *Disruptive scenarios analysis* A what-if analysis should be performed for each of the previous scenarios defined. To implement a specific scenario, it is necessary to modify the input data corresponding to the disruptions under study and re-run the SD model. At this point, the SD model: (a) provides a solution to cope with the level of disruptions contemplated in each SC scenario and (b) allows to perform a what-if analysis including the observation of the AFSC operation (new solution), its associated key performance indicators (usually in terms of the SD model objectives) and the calculation of the robustness indicators.
4. *Assessment of the SC robustness for each scenario* The calculated value of the robustness indicators for each scenario is checked to be inside their allowed thresholds (limits). It can be considered that the AFSC is not robust for the scenarios in which the thresholds of one or more robustness indicators are not respected.
5. *Definition, evaluation and selection of protective actions* If a SC is found to lack robustness to certain levels of disruptions or combination of them, risk protection actions should be evaluated to improve its robustness. Once the potential proactive actions are defined and implemented in the model by changing its input data, the SD model is executed providing the new SC robustness as well as other SC performance indicators. Based on these indicators, the most satisfactory protective actions can be selected. At this point, it is important to note that the SD model provides the SC not only with the performance indicators when implementing the proactive actions but also with the solution for the SC operation in case a disruption will occur and the protective action will be implemented.

## Application of the methodology to an AFSC

To validate the proposed methodology, the designed AFSC and the planting planning reported in Esteso et al. (2021) that optimizes the SC profits for products with a limited shelf-life has been considered.

### Robustness indicators definition and their thresholds

For this research, the robustness of the AFSC is measured through the profit per kilogram of vegetables sold (hereinafter referred to as unitary profit). The AFSC is considered to be robust if the unitary profit is higher than the threshold of 0.1 €/kg.

### Disruptive scenarios identification

In our case, four types of disruptions are identified with different levels. Based on them, 46 scenarios in total have been identified: one baseline scenario, 44 scenarios representing disruptions in demand, supply, transport between SC nodes, and SC nodes operability and one more scenario combining different disruption types (Table 5).

**Table 5:** SC Robustness assessment scenarios

Disruption type	Scenario	Description
None	BS	Baseline scenario
Demand	0.50D	Demand is 50% of demand in the baseline scenario
	0.75D	Demand is 75% of demand in the baseline scenario
	1.25D	Demand is 125% of demand in the baseline scenario
	1.5D	Demand is 150% of demand in the baseline scenario
	1.75D	Demand is 175% of demand in the baseline scenario
	2D	Demand is 200% of demand in the baseline scenario
Supply	0.50Y	Plant yield is 50% of the yield in the baseline scenario
	0.75Y	Plant yield is 75% of the yield in the baseline scenario
	1.25Y	Plant yield is 125% of the yield in the baseline scenario
	1.5Y	Plant yield is 150% of the yield in the baseline scenario
Transport	F1-P1	Not possible to transport vegetables between farm 1 and PP 1
	F2-P1	Not possible to transport vegetables between farm 2 and PP 1
	F3-P1	Not possible to transport vegetables between farm 3 and PP 1
	F4-P1	Not possible to transport vegetables between farm 4 and PP 1
	F5-P1	Not possible to transport vegetables between farm 5 and PP 1
	F2-P2	Not possible to transport vegetables between farm 2 and PP 2
	F3-P2	Not possible to transport vegetables between farm 3 and PP 2
	F4-P2	Not possible to transport vegetables between farm 4 and PP 2
	F5-P2	Not possible to transport vegetables between farm 5 and PP 2
	F4-P3	Not possible to transport vegetables between farm 4 and PP 3
	F5-P3	Not possible to transport vegetables between farm 5 and PP 3
	P1-D1	Not possible to transport vegetables between PP 1 and DC 1
	P2-D1	Not possible to transport vegetables between PP 2 and DC 1
	P3-D1	Not possible to transport vegetables between PP 3 and DC 1
	P3-D2	Not possible to transport vegetables between PP 3 and DC 2
	D1-M1	Not possible to transport vegetables between DC 1 and market 1
	D1-M2	Not possible to transport vegetables between DC 1 and market 2
	D1-M3	Not possible to transport vegetables between DC 1 and market 3
	D1-M4	Not possible to transport vegetables between DC 1 and market 4
	D2-M1	Not possible to transport vegetables between DC 2 and market 1
	D2-M2	Not possible to transport vegetables between DC 2 and market 2
	D2-M3	Not possible to transport vegetables between DC 2 and market 3
	D2-M4	Not possible to transport vegetables between DC 2 and market 4
Operability of SC nodes	F1	Farm 1 become inoperative
	F2	Farm 2 become inoperative
	F3	Farm 3 become inoperative
	F4	Farm 4 become inoperative
	F5	Farm 5 become inoperative
	P1	PP 1 become inoperative
	P2	PP 2 become inoperative
	P3	PP 3 become inoperative
	W1	Warehouse 1 become inoperative
	D1	DC 1 become inoperative
	D2	DC 2 become inoperative
Mixed scenario	1.25D + F2 + F5	Demand is 125% of demand in the baseline scenario, F2 and F5 become inoperative

### Disruptive scenarios analysis and assessment of the AFSC robustness for each scenario

At this step the implementation of the above scenarios in the SD model is made by changing its input data. Based on the new data, the SD model is re-run providing the impact of the different disruption types and their level on the SC performance and its robustness which is compared with the so called “baseline scenario” with no disruptions that represents the benchmark. In the following more detail is provided.

#### Baseline scenario

The data used in the baseline scenario have been extracted from Esteso et al. (2021), in which fresh vegetable supply chains are designed considering the product perishability. Specifically, the AFSC was designed to market three vegetables with a shelf life equivalent to five weeks, resulting in five farms, three PPs, one warehouse, two DCs, and four markets. The planning horizon is one year divided into 52 weekly periods, with the first period corresponding to the first week of July, when the planting season for the vegetables begins.

The farms have a planting available area of 190, 210, 170, 130 and 290 hectares respectively. Their capacity to plant, cultivate and harvest vegetables is limited by the labourers on staff, and it is possible to hire and fire temporary and permanent labourers at a weekly cost of 42.5 and 69 € respectively. The cost of hiring permanent labourers is equivalent to one month’s salary. Farms must hire at least one permanent labourer for every 10 hectares available. Labourers work 2,800 min per week, and each labourer needs 2,800 min to plant one hectare of vegetable, 752 min per week to cultivate one hectare, and between 1,500 and 3,000 min to harvest one hectare depending on the vegetable planted.

The planting plan in each of the farms is known (Table 6). The weekly yield of the plants depends on the planting and harvesting period ranging between 3,080 and 14,520 kg/ha for vegetable A, 2,860 and 12,760 kg/ha for vegetable B, and 380 and 3,420 kg/ha for vegetable C.

**Table 6:** Planting plan

Farm	Vegetable	Planting period				
		3	5	18	27	31
Farm 1	Veg A		33.36 ha	17.52 ha	68.67 ha	
	Veg B		23.94 ha	10.66 ha	20.78 ha	
	Veg C	10.02 ha				
Farm 2	Veg A	16.65 ha	51.33 ha	50.75 ha		17.32 ha
	Veg B		28.45 ha		32.50 ha	
	Veg C		6.79 ha	6.22 ha		
Farm 3	Veg A	13.25 ha	50.33 ha	18.31 ha	40.58 ha	
	Veg B		26.20 ha		11.46 ha	
	Veg C			9.85 ha		
Farm 4	Veg A		19.96 ha	31.96 ha	0.18 ha	19.29 ha
	Veg B	10.31 ha				2.36 ha
	Veg C		19.49 ha			7.56 ha
Farm 5	Veg A	17.28 ha	51.49 ha		24.74 ha	48.39 ha
	Veg B		36.32 ha	28.48 ha	18.88 ha	25.54 ha
	Veg C	5.72 ha		11.19 ha	5.43 ha	6.23 ha

Each PP has a processing capacity of 1,800,000 kg per week, the warehouse has a handling capacity of 19,200,000 kg per week and a storage capacity of 3,600,000 kg, while each DC have a handling capacity of 4,800,000 kg per week and a storage capacity of 240,000 kg.

The overall weekly demand ranges between 2,600,000 and 3,800,000 kg for vegetable A, 1,150,000 and 1,650,000 kg for vegetable B, and 125,000 and 185,000 kg for vegetable C, the weekly average being approximately 3,100,000 kg, 1,345,000 kg, and 150,000 kg respectively. Economic data (price of vegetables, unitary holding costs, transport costs, etc.) can be found in Esteso et al. (2021).

After running the SD model for the baseline scenario, profits of 19,083,450 € are obtained as the difference between sales (56,785,564 €) and costs related to the opening of AFSC nodes (14,225,200 €), the planting, cultivating and harvest of vegetables (1,919,081 €), the hiring and salaries of labourers (809,884 €), the packing of vegetables (4,034,924 €), their storage (448,647 €), handling (2,689,728 €) and transport (7,807,829 €), and the economic penalties for the waste of vegetables (304,740 €) and unmet demand (5,462,081 €).

**Fig. 7:**
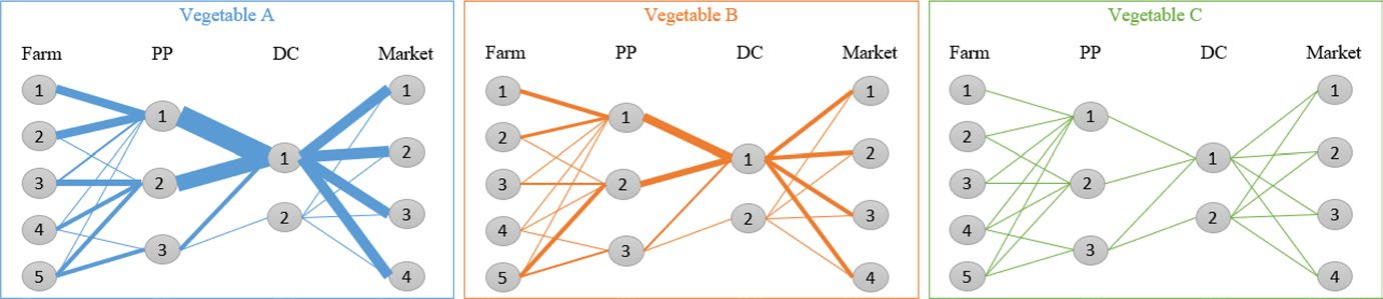
Vegetables flow in the baseline scenario

Figure 7 shows the flow of vegetable A, B and C through the AFSC, showing the connections used between the different nodes of the chain. The thickness of the arc represents the amount of vegetable transported through them, so most of the traded product passes through PPs 1 and 2 as well as DC 1. It should be noted that the warehouse available between the PPs and the DCs is not used in the baseline scenario.

In addition, the SD model provides decision-makers with information related to the main decisions to be taken weekly by each of the nodes. In the farms, the area to be cultivated, and harvested on a weekly basis is shown, as well as the quantity of each vegetable to be harvested and transported to each PP in each week, and the quantity of permanent and temporary labourers to hire and fire per week. For the PPs, the SD model shows the quantity of each vegetable to be received weekly from the farms, and the quantity to be packed, stored, wasted, or transported to each DC and warehouse. For the warehouse, the quantity of each vegetable to be received from the PPs on the weekly basis and the quantity to be handled, stored, wasted or sent to DCs weekly is shown. For the DCs, the quantity of each vegetable received from the PPs and warehouses per week is shown, as well as the quantity to be handled, stored, wasted or sent to each market. Finally, for the markets show the quantity of each vegetable received from the DCs, and the quantity of each vegetable to be stored, wasted, sold per week as well as the unmet demand for each vegetable.

As an illustrative example, the Appendix shows the decisions made in the baseline scenario for farm 1 (Fig. 13), PP 1 (Fig. 14), DC 1 (Fig. 15) and market 1 (Fig. 16).

#### Disruption in demand

This section analyses how the tool can be used to measure the robustness of the AFSC to scenarios representing demand disruptions. An example of such potential demand disruptions is the occurred during the first month of the COVID pandemic lockdown, where some basic agri-food products saw their demand increased by 90% over the previous year (Kalogiannidis and Melfou 2020) while others with a very short shelf-life saw their demand drastically reduced to the detriment of an increase in demand for frozen or longer shelf-life vegetables (Charlton and Castillo 2021).

Since this model allows the robustness of the planting planning carried out in the SC to be assessed, the possibility of varying the area to be planted of each vegetable to adjust production to the demand defined in each scenario is not considered.

**Fig. 8:**
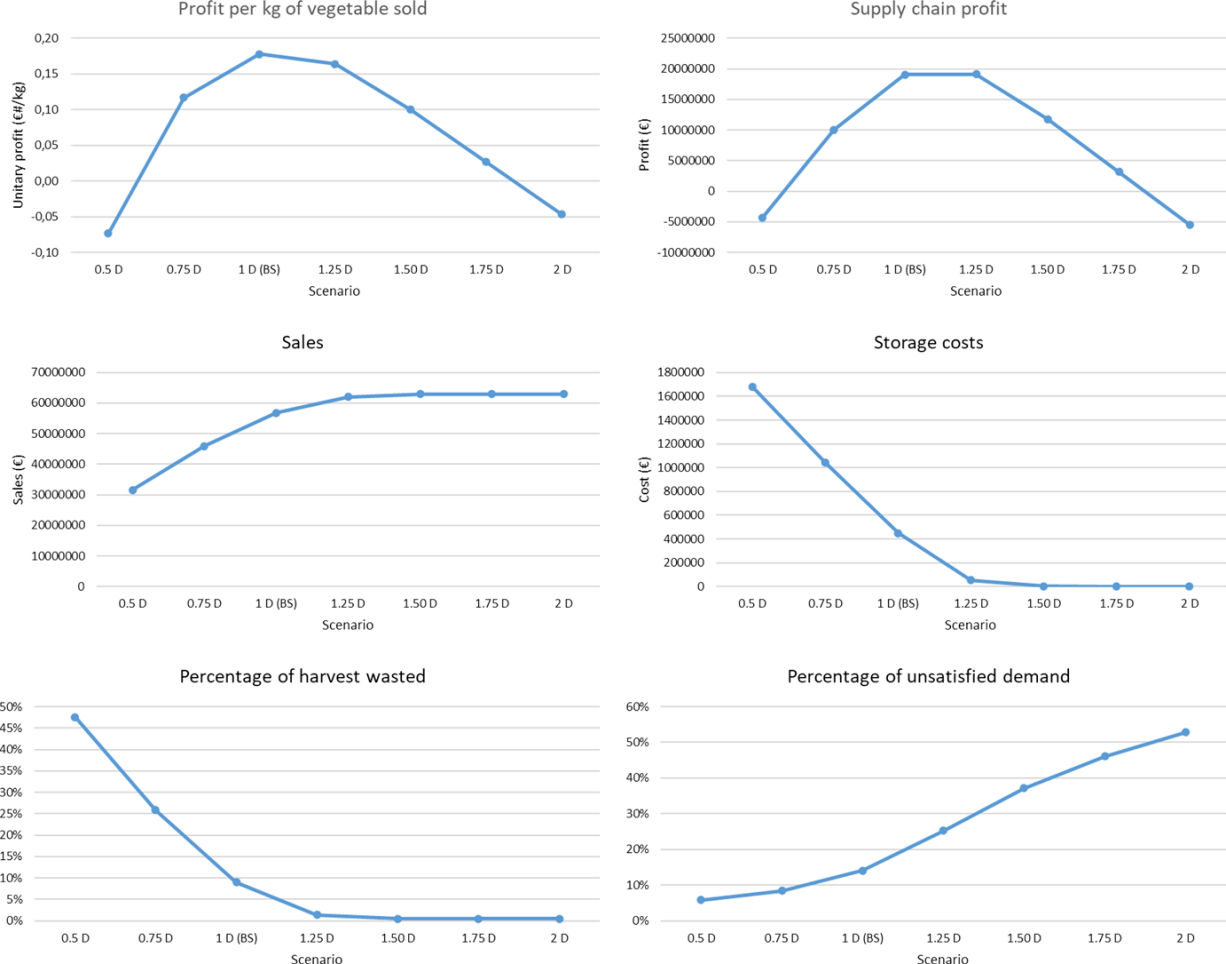
Results of scenarios with demand disruption

Figure 8 presents in addition to the profit per kilogram of vegetable sold, the SC results that are affected by the variation in demand, which are SC profit, sales, storage costs, percentage of wasted vegetables and percentage of unsatisfied demand.

The analysis of the unitary profit shows that, if the SC members set the limit for this indicator at 0.1 €/kg, the SC and its planting plan would be robust in scenarios where demand is between 75 and 150% of the baseline scenario.

The reduction in total SC profits in the scenarios where demand is reduced is due to the loss of sales as well as the increase of the costs of storing harvested and packed vegetables that cannot be sold due to the shortage of demand and the economic penalty for wasting excess vegetable production. On the other hand, the reduction in total SC profits in scenarios where demand increases is mainly due to the unsatisfied demand penalty of not having enough product to satisfy demand. It is remarkable that in these scenarios inventory costs and wastage are reduced until they become non-existent and that sales remain stable as they are limited by the quantity of vegetables harvested.

It is concluded that the SC under consideration is not robust in scenarios where demand is substantially varied. To increase robustness to such disruptions, protective actions could be taken such as buying vegetables from other regions in cases where demand increases by more than 50% or selling excess vegetables to other markets such as composting or processed agri-food products in cases where demand falls by more than 25%.

#### Disruption in supply

This section analyses how the tool can be used to measure the robustness of the AFSC to scenarios where supply is reduced or increased. In this case it is analyzed what would happen if the yield of the plants were different than expected because of some disruption such as flooding, drought, etc. The possibility of varying the area to be planted of each vegetable to adjust production to the demand is not considered since this section assesses the robustness of the planting planning carried out in the SC.

**Fig. 9:**
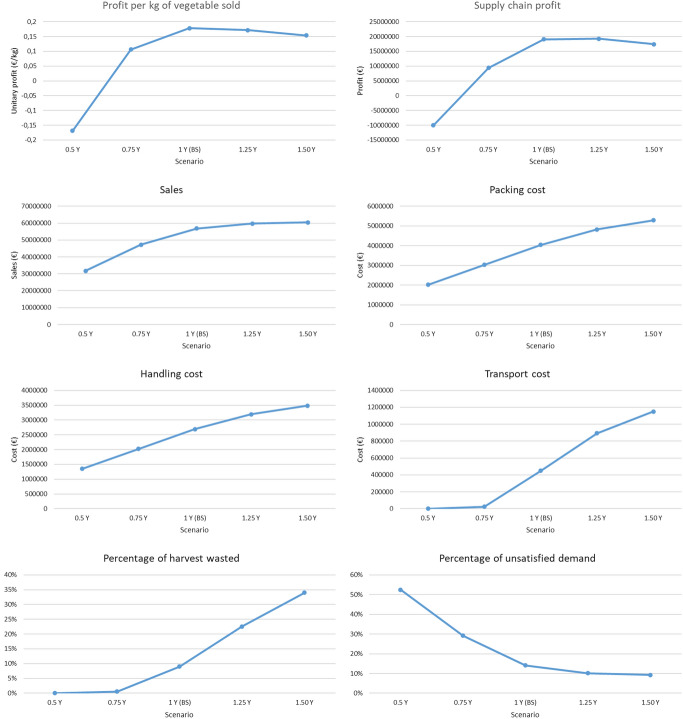
Results of scenarios with supply disruption

Figure 9 presents in addition to the profit per kilogram of vegetable sold, the SC results that are affected by the variation in the yield of the plants, which are SC profit, sales, packing costs, handling costs, transport costs, percentage of wasted vegetables and percentage of unsatisfied demand.

If members of the SC were to set the limit for unitary profit at 0.1€/kg, the SC would be robust to all the supply disruptions analyzed except for the scenario in which plants obtain 50% of the yields considered in the baseline scenario.

The total SC profits are drastically reduced in the scenarios where plant yield is lower than in the baseline scenario because the decrease in sales and the increase in the economic penalty for unsatisfied demand outweighs the reduction in costs related to packing, handling, and transport.

On the other hand, the SC profits decrease in scenarios where plant yield is higher than in the baseline scenario, as the costs related to packing, handling, transport, and economic penalties for waste are higher than the increase in sales. This is mainly due to the limited capacity of the packing plants, which once saturated prevent the harvested vegetables from being packed and sent to the markets to satisfy the unsatisfied demand. Therefore, despite the increase in vegetable harvest, the percentage of wasted harvest increases considerably while the percentage of unsatisfied demand remains constant.

It is concluded that the SC under consideration is not robust in cases where plant yields decrease by more than 25%. To increase the robustness of the SC in these cases, vegetables could be purchased from other regions not affected by the disruption. On the other hand, facility capacity has been found to act as a bottleneck in scenarios where plant throughput increases, a problem that could be solved by increasing facility capacity or by opening new facilities in the SC.

#### Disruption in transport

**Fig. 10:**
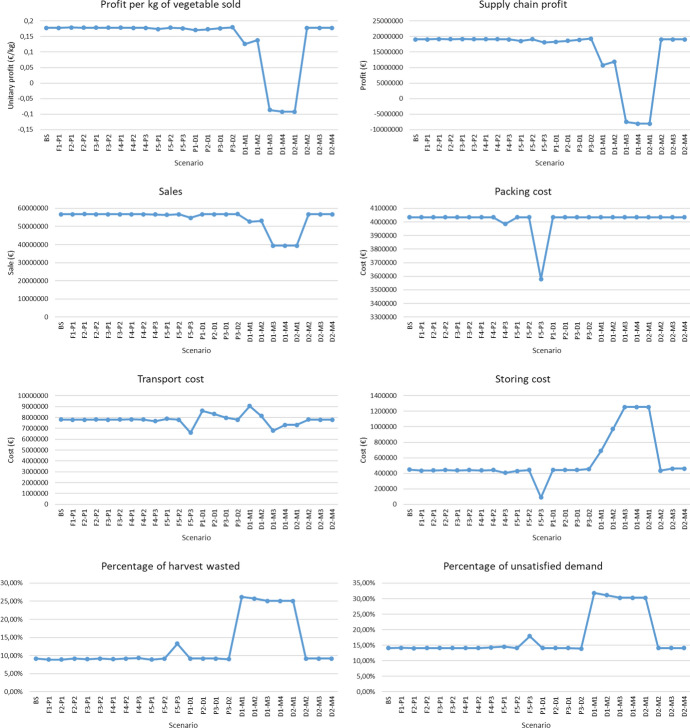
Results of scenarios with supply disruption

This section discusses how the proposed tool can be used to measure the robustness of the AFSC to scenarios where it is not possible to transport vegetables between two SC nodes due to transport disruptions. Figure 10 presents the profit per kilogram of vegetable sold and the SC results that affected by these disruptions: SC profit, sales, packing costs, transport costs, storage costs, percentage of wasted vegetables and percentage of unsatisfied demand.

If the SC members were to set the limit for the unitary profit at 0.1€/kg, the SC would be robust for all scenarios with transport disruptions between two nodes of the SC except for those where it is not possible to transport vegetables between DC1 and markets 3 or 4 or between DC2 and market 1 since all vegetables transported to markets 3 and 4 come from DC1 and vegetables that reach market 1 do so mainly via DC2.

In these cases, less vegetables reach the disrupted markets, thus reducing sales, SC profits, and therefore reducing the percentage of unsatisfied demand. Furthermore, as this product cannot be shipped to another market, it is stored in DCs, thus increasing their costs, and eventually goes to waste, increasing the percentage of wasted crop.

In the remaining scenarios, the SC profits remain stable as the model decides to transport the vegetables through different arcs than the ones suffering the disruption, thus ensuring that the harvested vegetables reach the markets. Therefore, small differences in transport and storage costs are observed due to the use of different transport arcs than those used in the baseline scenario.

It is concluded that the SC is robust to all the transport disruptions analyzed except those between DC1 and markets 3 and 4, and between DC2 and market 1. To increase the robustness of the SC and minimize the impact of these disruptions, alternative modes of transport should be considered or markets could be forced to purchase vegetables from more than one DC or from other suppliers outside the SC.

#### Disruption in the operability of SC nodes

**Fig. 11:**
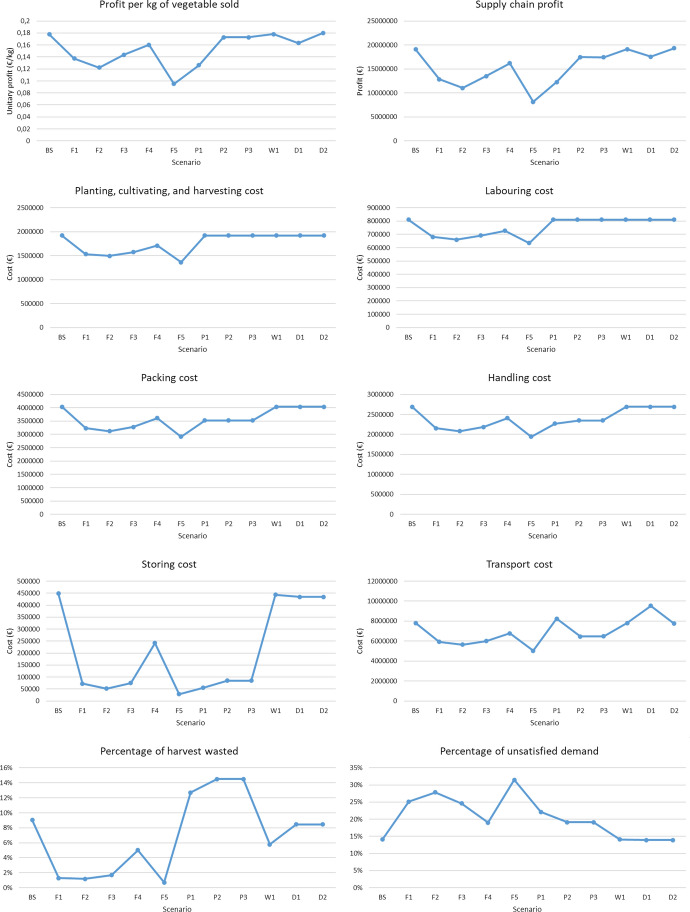
Results of scenarios with node operability disruption

This section analyses how the proposed tool can be used to measure the robustness of the AFSC to scenarios where a node becomes inoperative due to disruptions. These disruptions can be caused by multiple reasons such as the lockdown of the country or the region where it is located (as happened with the COVID pandemic) or a fire or flooding of facilities, among others. Figure 11 presents in addition to the profit per kilogram of vegetable sold, the SC results that are affected by the disruptions in the operability of SC nodes, which are SC profit, planting, cultivating, and harvesting costs, laboring costs, packing costs, handling costs, storing costs, transport costs, percentage of wasted vegetables and percentage of unsatisfied demand.

If SC members set the limit value of unitary profit at 0.1€/kg, the SC analyzed would be robust to disruptions in the operation of SC nodes. However, these disruptions generate large variations in SC profits.

The costs related to the agricultural stage (planting, cultivating, and harvesting costs, and laboring costs) are only modified when one of the farms becomes inoperative, with the impact on the SC being different between farms since, according to the baseline scenario they provided with different quantity of vegetables to the SC.

If one PP becomes inoperative, the capacity of the SC to pack and market vegetables is reduced. This is because by working with one PP less, the operative plants use all their capacity, becoming bottlenecks in the SC. Therefore, the flow of vegetables through the SC is reduced and the quantity of wasted harvested vegetables is increase.

The inoperability of the warehouse has no effect on the SC since in the baseline scenario the warehouse is not used at any point in the simulation. Therefore, the SC would be fully robust to any disturbance related to the warehouse.

Finally, the performance of the SC is very similar to that of the baseline scenario when one of the DCs become inoperative. This is because the baseline scenario does not use the full capacity of the DCs, so if one becomes inoperative the other is able to carry out the handling, storage and transport of the vegetables coming from the PPs.

It is concluded that the SC analyzed is robust to disruptions in the operability of the SC nodes, since in all the scenarios considered a unit profit equal to or greater than 0.1 €/kg. However, if the robustness of the SC is to be increased, new nodes could be selected to be part of the SC.

#### Multiple disruptions in demand and operability of SC nodes

**Fig. 12:**
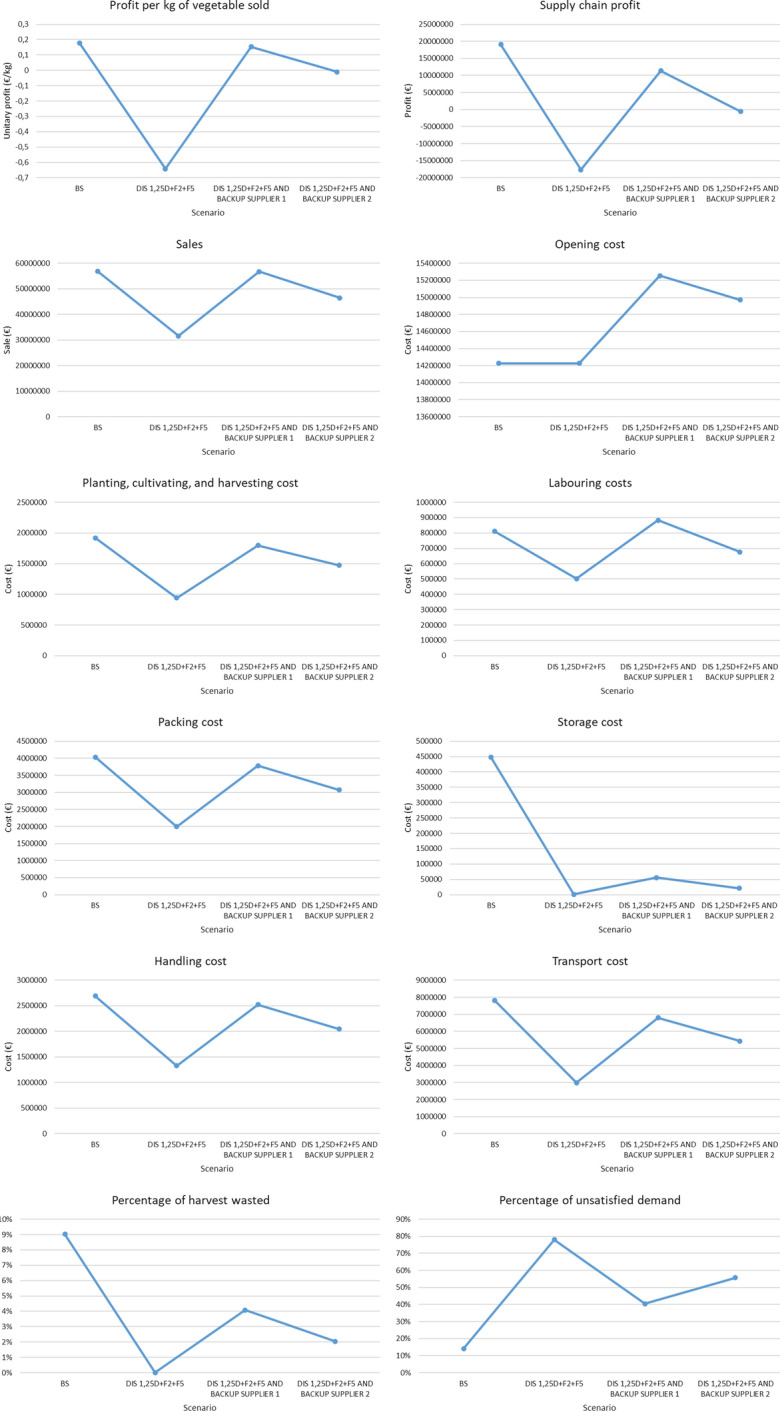
Results of scenarios with possible proactive actions

**Fig. 13:**
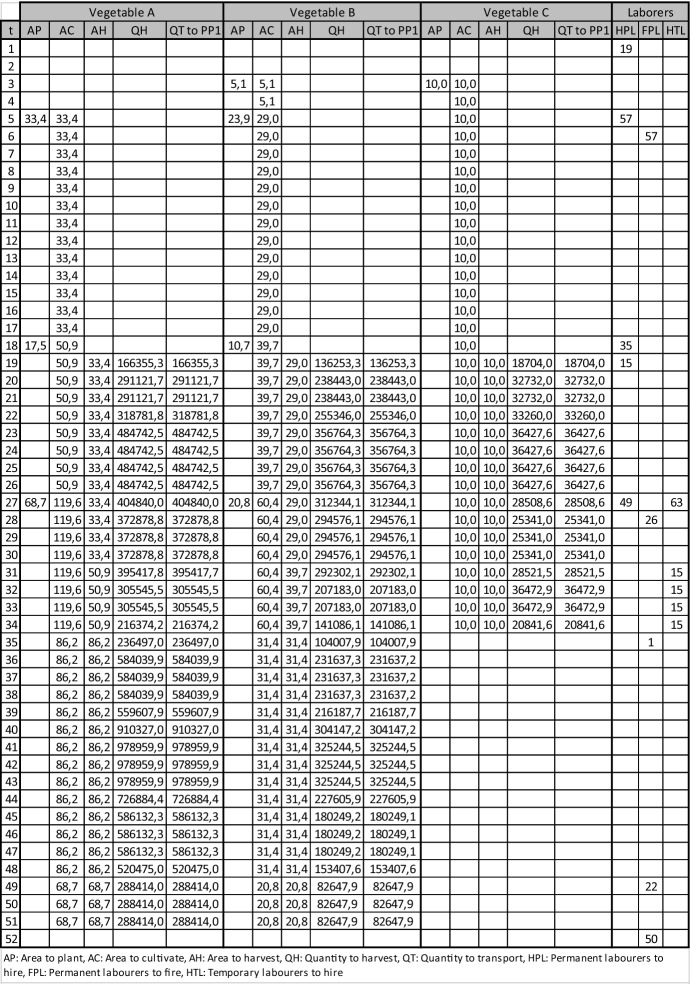
Farm 1 decisions in the baseline scenario

**Fig. 14:**
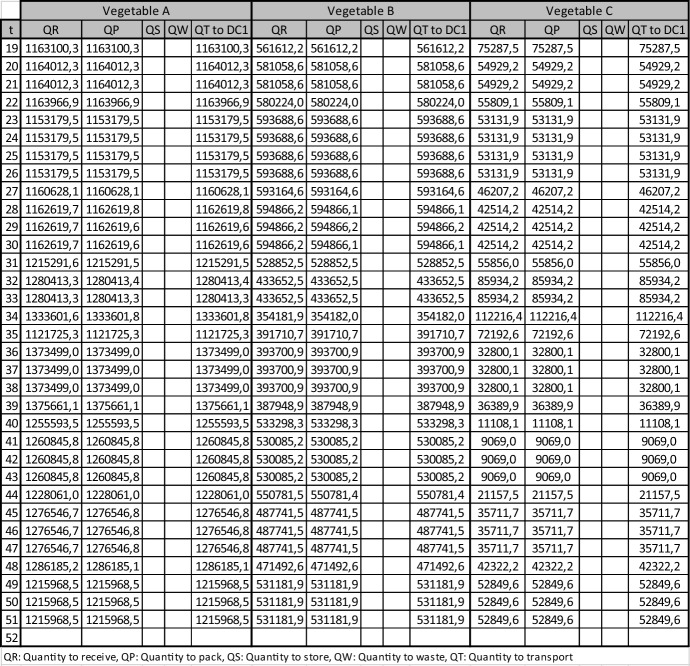
PP1 decisions in the baseline scenario

**Fig. 15:**
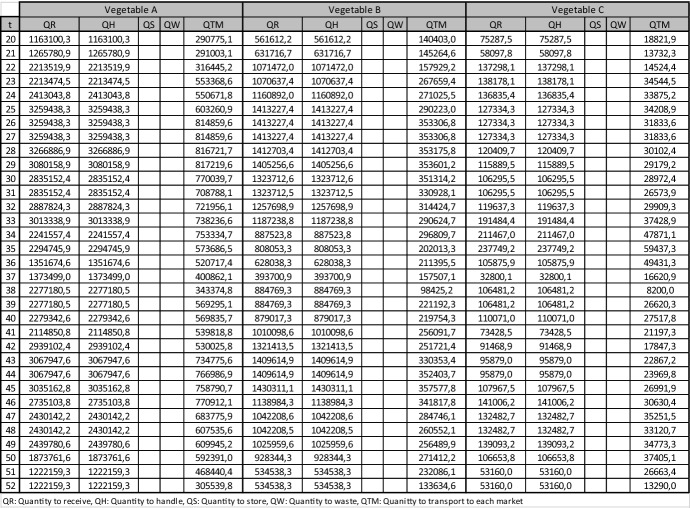
DC 1 decisions in the baseline scenario

**Fig. 16:**
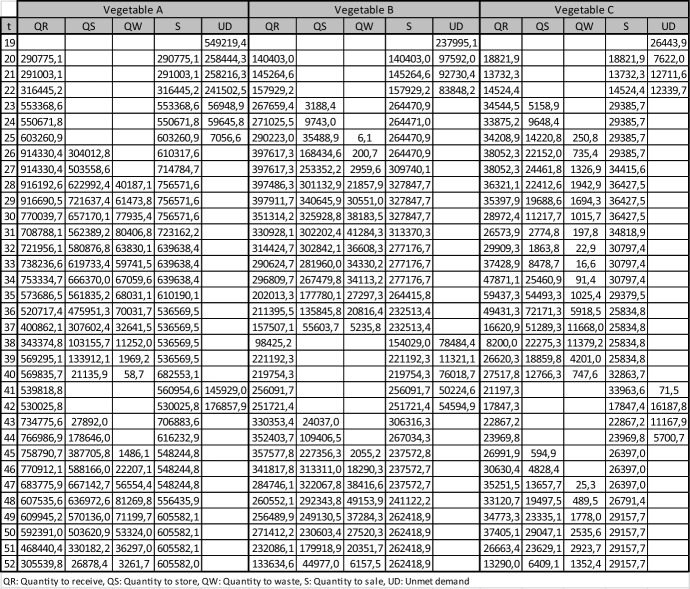
Market 1 decisions in the baseline scenario

This scenario aims to show the utility of the SD model to address the analysis in case multiple disruptions types simultaneously occur. For the AFSC under study, it is assumed that a high probability exists of an increase of 25% in demand (1.25D) and farmers F2 and F5 become inoperative at the same time. In the event that this scenario occurring, the robustness indicator (the profit per kg of vegetable sold) takes the value of −0.642 €/kg, falling sharply below the threshold for robustness (0.1 €/kg) (Fig. 12). This indicates that the AFSC is extremely vulnerable to this combination of disruptions, being necessary to protect it with proactive actions. So, following with the methodology proposed, risk protection actions should be defined that are detailed in the next subsection. The other studied performance indicators can be also consulted in Fig. 12.

### Definition, evaluation, and selection of risk protection actions

In the event that the SC has shown to be robust to the disruptions from which its members want to be protected, the SC could continue to operate as usual without taking any action to protect against the risk. However, if the SC proves not to be robust to the disruptions it wishes to be robust to, actions can be taken to increase its robustness. In our case, due to the high probability of the multiple scenario disruption to occur and in order to avoid such a sharp drop in the SC indicators, a protective action that modifies the original AFSC design is considered: the selection of a backup supplier. Two possible backup suppliers with different planted areas for each vegetable, transportation characteristics and opening costs are analyzed.

The backup supplier 1 provides a planting area of 450 ha with the planting plan of Table 7. Its opening cost amounts to 472,120 €. The transport time from it to packing plants are: one week to PP1, two weeks for PP2 and two weeks for PP3.

**Table 7:** Planting plan of backup supplier 1

Vegetable	Planting period					
	3	5	14	18	27	31
Vegetable A	40 ha	60 ha	20 ha	60 ha	–	30 ha
Vegetable B	–	60 ha	30 ha	–	50 ha	10 ha
Vegetable C	30 ha	20 ha	40 ha	–	–	–

The backup supplier 2 presents a planting area of 289 ha with the planting plan of Table 8. Its opening cost amounts to 341,880 €. The transport time from it to packing plants are: one week to PP1, two weeks for PP2 and two weeks for PP3.

**Table 8:** Planting plan of backup supplier 2

Vegetable	Planting period					
	3	5	14	18	27	31
Vegetable A	20 ha	50 ha	–	–	20 ha	30 ha
Vegetable B	–	40 ha	–	10 ha	20 ha	30 ha
Vegetable C	10 ha	–	–	30 ha	10 ha	10 ha

After defining risk protection actions, a new robustness analysis is performed using the SD model to determine whether the action taken has had a positive impact on the robustness of the SC or not. For the case under study, both protective actions improve the robustness of the AFSC as compared to remain impassive in the face of the multiple disruption (DIS 1,25D + F2 + F5) as shown in Fig. 12. However, only the backup supplier 1 restore the robustness of the AFSC since the profit per kg of vegetable sold (0.153 €/kg) is higher than the threshold defined (0.1€/kg). This is not the case for the protective action of choosing backup supplier 2, whose value (−0.01€/kg) still remains lower than the desirable value (0.1€/kg). Both backup suppliers improve the SC profit compared to the no action scenario, although being the improvement more pronounced for the backup supplier 1. Even for this backup supplier the SC profit does not achieve that of the baseline scenario (BS). The main differences between these two scenarios are the additional cost incurred in opening the backup supplier 1 and the volume of waste and unsatisfied demand. The planted area in the BS scenario leads to more harvested quantities than the scenario with the backup supplier 1. This results in lower percentages of unsatisfied demand but at the same time, in higher vegetable waste due to the vegetables perishability and the mismatch in consumption (demand) and yield patterns of plants originated. After analysing the three possible courses of actions (not to take any action, to select the backup supplier 1 or backup supplier 2), the backup supplier 1 is selected in order to protect the AFSC to the studied disruption scenario.

## Conclusions and future research lines

This paper has proposed a SD model to analyze the robustness of the fresh AFSC configuration and its planting planning to disruptions in demand, supply, transport between SC nodes, and the operability of SC nodes. The model is validated through the extreme conditions test and the known behavior reproduction test. In order to show how to use the SD model for improving the SC robustness, a methodology is proposed. This methodology is validated through its application to a case study and solved for a baseline scenario and 45 scenarios representing the types of disruptions to determine the robustness of the AFSC under study.

Since the robustness of the SC is a subjective element that depends on the risk aversion of its members, this paper measures the robustness of the SC through the profit per kilogram of vegetables sold and sets its limit value at 0.1 €/kg, considering that the SC is robust when the value of the unitary profit is higher than this limit.

After experimentation, it is concluded that the AFSC configuration and its planting planning is robust to 80% of disruptions analyzed, which are 30% of the demand disruptions, 75% of the supply disruptions, 85% of the transport disruptions, and 91% of the disruptions in the operability of the SC. A mixed scenario with a combination of different types of disruptions is analyzed. The analysis reveals a pronounced lack of SC robustness against this scenario. For this reason, a protective action is defined consisting in the redesign of the AFSC by incorporating one backup supplier from among two possibilities. The execution of the SD model with each of this two backup suppliers allows to know in advanced, the level of protection provided by each one supplier to the AFSC based the different robustness indicators. For our case, the backup supplier 1 is chosen as a proactive course of action.

It should be noted that these conclusions cannot be extrapolated to other AFSCs but are specific to the AFSC analyzed in this article. In order to analyze the robustness of another AFSC to such disruptions, the proposed methodology should be applied and the model would have to be run by changing the input data and adapting it to the functioning of the new SC.

Besides, the proposed model could be employed for different uses that the one proposed in this paper. It could be used to analyze the robustness of the AFSC configuration and its planting planning to disruptions in demand, supply, transport, and operability of nodes different from those proposed in this paper. This would require modifying inputs related to demand (demand disruptions), plant yield or planted area (supply disruptions), arcs between which it is possible to transport product (transport disruptions) and operative nodes (SC node operability disruption). Additionally, scenarios combining more than two types of disruptions could be envisaged.

The model could also be used to analyze the robustness of SC decisions different than SC configuration and planting planning. For this purpose, the values of the decisions to be assessed should be included as inputs to the model. For example, to assess the robustness of the packing planning to certain disruptions, the values of the decisions related to packing should be set as model inputs and the model run for the desired disruptive scenarios.

The model can be used to analyze the SC robustness to other disruptions, such as a disruption of the processing or storage capacity of one or more nodes in the SC. This would only require considering different “what-if” scenarios in which different values are assigned to those elements of the SC that could be modified by the disruption (e.g., the storage capacity of a DC).

Finally, the model not only serves to analyze the impact of disruptions on SC performance but could also be used to analyze different policies for the transport, packing, or inventory of vegetables or to analyze the impact of uncertainty on different factors such as the shelf-life of vegetables or transport time between the SC nodes. To test the impact of a change in a policy decision on the SC outcomes, the decisions related to the policies should be changed and the model re-run, and it would be possible to compare the results obtained for the different policies analyzed.

All these proposals for possible uses of the presented model could be tested and validated in future studies. Furthermore, as a future line of research, the proposed model could be extended to assess not only the robustness of the AFSC but also its resilience, which is the capacity of a SC to recover after a disruption.

## Appendix

See Figs. 13, 14, 15, 16.
